# APASL consensus statements and recommendation on treatment of hepatitis C

**DOI:** 10.1007/s12072-016-9717-6

**Published:** 2016-04-29

**Authors:** Masao Omata, Tatsuo Kanda, Lai Wei, Ming-Lung Yu, Wang-Long Chuang, Alaaeldin Ibrahim, Cosmas Rinaldi Adithya Lesmana, Jose Sollano, Manoj Kumar, Ankur Jindal, Barjesh Chander Sharma, Saeed S. Hamid, A. Kadir Dokmeci, Mamun-Al-Mahtab, Geofferey W. McCaughan, Jafri Wasim, Darrell H. G. Crawford, Jia-Horng Kao, Osamu Yokosuka, George K. K. Lau, Shiv Kumar Sarin

**Affiliations:** 1 Yamanashi Prefectural Central Hospital, 1-1-1 Fujimi, Kofu-Shi, Yamanashi 400-8506 Japan; 2 The University of Tokyo, 7-3-1 Hongo, Bunkyo-ku, Tokyo, 113-8655 Japan; 3 Graduate School of Medicine, Chiba University, Chiba, Japan; 4 Peking University Hepatology Institute, Peking University People’s Hospital, Beijing, China; 5 Kaohsiung Municipal Ta-Tung Hospital, Kaohsiung, Taiwan; 6 Hepatobiliary Division, Department of Internal Medicine, Kaohsiung Medical University Hospital, Kaohsiung Medical University, Kaohsiung, Taiwan; 7 GI/Liver Division, Department of Internal Medicine, University of Benha, Benha, Egypt; 8 Digestive Disease and GI Oncology Center, Medistra Hospital, University of Indonesia, Jakarta, Indonesia; 9 University Santo Tomas Hospital, Manila, Philippines; 10 Department of Hepatology, Institute of Liver and Biliary Sciences, New Delhi, India; 11 Department of Gastroenterology, G.B. Pant Hospital, New Delhi, India; 12 Department of Medicine, Aga Khan University and Hospital, Stadium Road, Karachi, 74800 Pakistan; 13 Department of Gastroenterology, Ankara University School of Medicine, Ankara, Turkey; 14 Department of Hepatology, Bangabandhu Sheikh Mujib Medical University, Dhaka, 1000 Bangladesh; 15 Centenary Institute, Royal Prince Alfred Hospital, University of Sydney, Sydney, Australia; 16 School of Medicine, University of Queensland, Woolloongabba, QLD 4102 Australia; 17 National Taiwan University College of Medicine and National Taiwan University Hospital, Taipei, Taiwan; 18 The Institute of Translational Hepatology, Beijing 302 Hospital, Beijing, China

**Keywords:** APASL, DAAs, HCV, Interferon-free, Turkey

## Abstract

The Asian-Pacific Association for the Study of the Liver (APASL) convened an international working party on the “APASL consensus statements and recommendation on management of hepatitis C” in March, 2015, in order to revise “APASL consensus statements and management algorithms for hepatitis C virus infection (Hepatol Int 6:409–435, 2012)”. The working party consisted of expert hepatologists from the Asian-Pacific region gathered at Istanbul Congress Center, Istanbul, Turkey on 13 March 2015. New data were presented, discussed and debated to draft a revision. Participants of the consensus meeting assessed the quality of cited studies. Finalized recommendations on treatment of hepatitis C are presented in this review.

**Electronic supplementary material:**

The online version of this article (doi:10.1007/s12072-016-9717-6) contains supplementary material, which is available to authorized users.

## Introduction

The major aim of antiviral treatment for chronic hepatitis C is to prevent liver-related complications, including HCC, by achievement of sustained virologic response (SVR) [1, 2]. The combination of peginterferon plus ribavirin can lead to ~50 and ~80 % SVR in patients infected with HCV genotype (GT)-1/GT-4 or HCV GT-2/GT-3, respectively [3]. In the direct-acting antivirals (DAAs) era, DAAs with peginterferon plus ribavirin can shorten the treatment duration and lead to ~90 % SVR in HCV GT-1-infected patients with interleukin-28B (IL28B, IFN-lambda 3) favorable single nucleotide polymorphism (SNP). But these treatments have many adverse events associated with interferon use which hamper the patients in accomplishing the treatments. Recently, interferon-free treatment has played a central role in the eradication of HCV. In this article, we aim to introduce the recent advances of interferon-free therapies for the patients with chronic HCV various genotypes in Asian-Pacific countries. Grading of evidence and recommendations are shown in Supplementary Table 1.

## Interferon supersensitive group

### Some viral and host factors are related to supersensitivity to the interferon-based therapy

HCV GT-2 is supersensitive to interferon-based therapy. The sustained virological response (SVR) rates with 24-week peginterferon and ribavirin treatment in chronic HCV GT-2 are around 80 % in western countries [4–8]. In the chronic HCV GT-2 trial from Taiwan, the SVR rates by treating with peginterferon and ribavirin can be higher than 95 % if the patients achieve a rapid virologic response (RVR) [9].

For chronic HCV GT-1 patients with RVR, high SVR rates, near 90 %, can be obtained by the peginterferon and ribavirin combination therapy [10]. The SVR rates were as high as 95 % by treating with peginterferon and ribavirin combination therapy in chronic HCV GT-1 patients with RVR and low viral load at baseline, even with the treatment duration shortened to 24 weeks [11–14]. The study by Atlanta Medical Center also demonstrated that the 24-week peginterferon and ribavirin regimen may have equal efficacy as a 28-week lead-in then boceprevir and peginterferon plus ribavirin triple therapy in chronic HCV GT-1 patients with low viral load and RVR [15].

Several genome-wide association studies have demonstrated that host SNPs near the IL28B gene are associated with SVR to the treatment with peginterferon alfa and ribavirin in chronic hepatitis C patients [16–18]. These SNPs are also associated with spontaneous clearance of HCV in acute HCV infection. The various distributions of IL28B polymorphisms among different populations worldwide may, at least partly, explain the heterogeneity in the responses to interferon-based treatments among different ethnic groups. The IL28B SNPs are strongly associated with SVR rates in patients who are infected with HCV GT-1 or GT-4 and receive combination treatment with peginterferon alfa and ribavirin [16–21]. However, the association between IL28B variations and treatment response in patients infected with HCV GT-2 or GT-3 is still controversial [22–24].

IL28B variations are associated with very early on-treatment viral kinetics in chronic hepatitis C patients who undergo interferon alfa-based therapy, and are the strongest pretreatment predictor of treatment response in patients infected with HCV GT-1 [19, 21–24]. Regarding the retreatment with peginterferon and ribavirin in chronic HCV GT-1 and GT-2 patients, the SVR rate for prior-relapser was quite good [25, 26]. However, the response was only found in the relapsers with favorable IL28B genotypes. Therefore, for the countries in which direct acting antiviral agents are available, generic drugs are not available, and only have limited resources, peginterferon and ribavirin combination therapy may be considered in chronic HCV GT-1, GT-4, or GT-6 treatment-naïve patients with low viral load and favorable IL28B genotypes and in HCV GT-2 or GT-3 naïve patients [27]. For the treatment-experienced patients, the peginterferon and ribavirin combination therapy can only be considered in relapsers with favorable IL28B genotype and non-cirrhotic patients.

### #1 Consensus statements and recommendation on interferon supersensitive group

1. For the countries in which direct acting antiviral agents are not available, or only have limited resources, peginterferon and ribavirin combination therapy may be considered in chronic HCV GT-1, GT-4, or GT-6 treatment-naïve patients with low viral load and favorable IL28B genotypes and in HCV GT-2 or GT-3 treatment-naïve patients. (A1)
2. For the treatment-experienced patients, the peginterferon and ribavirin combination therapy can only be considered in relapsers with favorable IL28B genotype and non-cirrhotic patients. (B2)

## Is interferon still needed in DAA affordable countries?

The development of direct acting antiviral agents and their inclusion in all-oral, interferon-free regimes has been the central element of the revolution in therapy for HCV infection. These new treatments are safe and very effective and there are virtually no medical reasons to withhold therapy [28]. Penetration of new interferon-free therapies into standard management plans in many countries in the Asian-Pacific region has been very slow despite outstanding responses to therapy in virtually every subgroup of patients treated to date [29, 30]. The delay in making these drugs available to patients is their high cost which is limiting uptake—irrespective of whether funding is patient-based or by government reimbursement. Restricted access to therapy is not limited to resource-restricted countries as many people living in countries with relatively high gross domestic products (GDP) still have limited availability. It is within this context that providers of health care are attempting to determine if there is a place remaining for the less expensive interferon-containing regimes in the emerging treatment paradigms.

Interferon has been a partner in the treatment of HCV for three decades and has played an important role in curing many patients and reducing their subsequent risk of cirrhosis or HCC [31]. Indeed, we have learnt much in the decades of experience gained using interferon-containing regimes. Optimising dosing regimens [5], PEGylation to improve pharmacokinetics [32], adding ribavirin [33] and discovering host genetic and other factors as well as viral characteristics that influence response [34] have all been major advances that enhanced treatment response rates. However, there are a number of disadvantages of interferon therapy that have limited its uptake and ensured that its role in antiviral therapy for HCV will eventually become largely obsolete. The side-effect profile and adverse effects of interferon therapy are substantial. In one report from Asia, up to 50 % of patients were judged to be unsuitable to commence therapy. Interferon-based therapy is lengthy—up to 1 year for genotype 1 patients—and requires substantial human and laboratory infrastructure to ensure safety, compliance and optimal dosing and treatment duration. The nature of the therapy limits its use to major treatment centers with only limited, if any, uptake in isolated areas. For these reasons, treatment uptake rates with interferon-based regimes have been modest, with some countries reporting treatment rates of only 5 % even when diagnosis rates were greater than 50 % [35].

In contrast, direct acting antiviral regimes are very well tolerated and treatment duration is shorter potentially falling to 8 weeks in some HCV populations [36]. Studies to date have shown outstanding treatment response rates across all genotypes, in previously treated and untreated subjects, pre and post liver transplant patients, HIV co-infected individuals and those with more advanced liver disease [37–42]. As stated earlier, there are virtually no medical reasons to withhold therapy. In this context it is not flippant to suggest that, at present, the only indication for interferon-based therapies is no access to direct acting antiviral therapies.

Arguments in favor of maintaining interferon therapies are that Asian patients respond reasonably well to these therapies, due in part to the enrichment of the population with the favorable IL28B allele. In HCV GT-1 Asian subjects, SVR of 75 % have been reported [43], with even more impressive responses in HCV GT-2 and GT-3 subjects. However, the issues that limit interferon uptake still exist as does on-treatment side effects and infrastructure requirements. Thus with current rates of therapy and continued use of interferon-based regimes, most countries in the Asian-Pacific region will continue on the inevitable path towards the peak of population morbidity and mortality predicted from HCV epidemiological studies and judged to be 10–20 years hence. Reducing the regional impact of this disease should be a major goal of governments in the Asian-Pacific region and this aim increases the urgency with which treatments should be made available. Of course, to have any impact on HCV-related disease burden, access to therapy must be increased. Without increasing access to care, the number of treated patients will not change. In the absence of such policies to increase access to direct acting antivirals, we are going to compound global inequalities in treatment and disease burden and individual suffering. The efforts by Gilead to assist in the provision of their medications to the 90 countries with the lowest GDP are to be congratulated. Global viral eradication was never going to be feasible with interferon-containing regimes. In contrast, there is a distinct possibility of eliminating HCV in the Asian-Pacific region with oral antiviral agents if sufficient attention is also paid to primary prevention.

## ALL-oral treatment for HCV GT-1 infection

In the Asian-Pacific region, except Australia, Iran, New Zealand, Philippines and Thailand, HCV GT-1b is the main subgenotype in HCV GT-1 [3]. During the interferon era, HCV GT-1 was the most intractable among all HCV GTs when patients were treated with peginterferon plus ribavirin [1]. Combined peginterferon plus ribavirin treatment for 48 weeks led to only ~50 % SVR in HCV GT-1 and high viral load. The addition of HCV NS3/4A protease inhibitors such as telaprevir, boceprevir or simeprevir to peginterferon plus ribavirin can improve SVR rates and shorten the treatment duration. These therapies can lead to 70–80 % and 90–100 % SVR in treatment-naïve and previous-treatment relapsers with HCV genotype 1b infection, respectively, but only ~30 % SVR in previous-treatment null responders [1, 44–48].

Furthermore, the combination of peginterferon plus ribavirin with or without protease inhibitor therapies usually results in various adverse events, which can occasionally be serious in certain patients despite those being treated with these therapies having been selected on the basis of SNPs of the IL28B gene and/or the inosine triphosphate pyrophosphatase (ITPA) gene [16–18, 49]. It is difficult to use peginterferon in interferon-ineligible/intolerant patients and it is hard to cure HCV-infected patients with unfavorable IL28B or with advanced liver fibrosis using interferon-including regimens [50, 51].

In 2010, interferon-free treatment for chronic HCV GT-1 infection was reported for the first time [52]. In this INFORM-1 trial, the oral combination of a nucleoside analogue polymerase inhibitor and protease inhibitor provided a proof-of-concept of “interferon-free treatment for chronic HCV GT-1” without treatment-related serious or severe adverse events [52]. New DAAs against HCV have since been developed, and these combinations without peginterferon can improve the SVR rates and shorten the treatment durations.

### Ledipasvir and sofosbuvir

#### Treatment-naïve HCV GT-1 patients

**Fig. 1:**
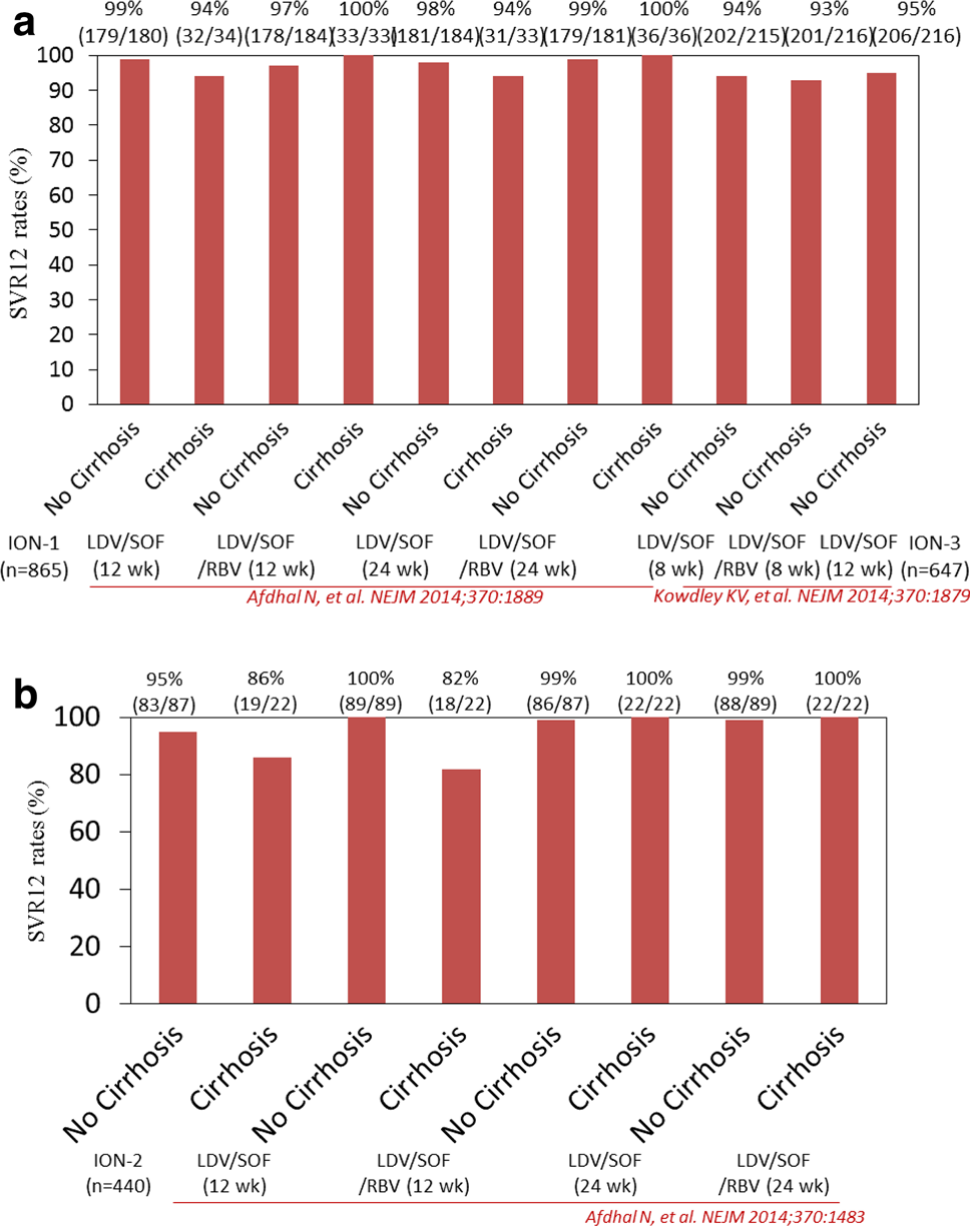
Sustained virological response (*SVR*) rates (%) of daily ledipasvir (*LDV*) and sofosbuvir (*SOF*). **a** Treatment-naïve patients with HCV genotype 1, **b** Treatment-experienced patients with HCV genotype 1

Ledipasvir is an HCV NS5A inhibitor with antiviral activity against HCV GT-1 [53]. Sofosbuvir is a nucleotide polymerase inhibitor with antiviral activity against HCV pan-genotypes [54]. After a phase 2 trial [54], an ION-1 study (*n* = 865) was conducted: a multicenter, randomized, open-label phase 3 trial for treatment-naïve HCV GT-1 patients with 12 or 24 weeks of a fixed-dose combination of ledipasvir (90 mg) and sofosbuvir (400 mg), with or without ribavirin (Fig. 1a) [30]. A total of 67 % of the patients had HCV GT-1a infection, 70 % had the non-CC IL28B (rs12979860) genotype, and 16 % had cirrhosis. Of the 865 patients, only 3 patients had virological failure: one with HCV GT-1b had virological breakthrough, while the other two, one with HCV GT-1a and one with HCV GT-1b, had virological relapse. Concerning HCV NS5A-resistant associated variants (RAVs), the relapser with HCV GT-1a had the L31M variant, and both patients with HCV GT-1b had the Y93H variant at the time of virological failure. Two of the relapsers also had HCV NS5A RAVs at baseline [30]. Treatment with 12 weeks of a fixed-dose combination of ledipasvir and sofosbuvir, with or without ribavirin, led to SVR 12 weeks after the end of treatment (SVR12) in 97 % (211/217) or 99 % (211/214) of the patients, respectively. Treatment with 24 weeks of a fixed-dose combination of ledipasvir and sofosbuvir, with or without ribavirin, led to SVR in 99 % (215/217) or 98 % (212/217) of the patients, respectively. Of the 33 patients who had a serious adverse event during treatment, 25 and 8 were in the 24- and 12-week regimens, respectively. Only 6 serious adverse events were observed, as follows: cellulitis, chest pain, gastroenteritis, hand fracture, non-cardiac chest pain and pneumonia, although fatigue, headache and nausea were the most common adverse events [54]. No patient in the 12-week group discontinued due to adverse events.

The ION-3 study (*n* = 647) consisted of a multicenter, randomized, open-label phase 3 trial for treatment-naïve HCV GT-1 patients without cirrhosis with 8 weeks of the combination of ledipasvir (90 mg daily) and sofosbuvir (400 mg daily), with or without ribavirin, or with 12 weeks of the combination of ledipasvir (90 mg daily) and sofosbuvir (400 mg daily) without ribavirin (Fig. 1a) [36]. A total of 80 % of the patients had HCV GT-1a infection and 75 % of the total patients had non-CC IL28B (rs12979860) genotype. Treatment with 8 weeks of the combination of ledipasvir and sofosbuvir, with or without ribavirin, led to SVR12 in 93 % (201/216) or 94 % (202/215) patients, respectively. Treatment with 12 weeks of combination of ledipasvir and sofosbuvir without ribavirin led to SVR12 in 95 % (206/216). Of the 23 patients who had a relapse, 15 had HCV NS5A RAVs at the time of relapse and 8 did not. Of these 15 relapsed patients, 9 had RAVs at baseline and 6 did not. Adverse events were more associated with ribavirin [36].

A Japanese study [55] also showed that treatment with 12-week treatment with the combination of ledipasvir (90 mg daily) and sofosbuvir (400 mg daily), with or without ribavirin, led to SVR12 in 96.4 % (80/83) or 100 % (83/83) of treatment-naïve HCV GT-1 patients, respectively. At present, sofosbuvir cannot be used in patients with estimated glomerular filtration rate (eGFR) ≦30 mL/min per 1.73 m^2^ or on hemodialysis.

#### Treatment-experienced HCV GT-1 patients

An ION-2 study (*n* = 440) has been carried out, consisting of a randomized, open-label phase 3 trial for HCV GT-1 patients who had not had SVR after treatment with peginterferon and ribavirin, with or without a protease inhibitor, with 12 or 24 weeks of a fixed-dose combination of ledipasvir (90 mg) and sofosbuvir (400 mg), with or without ribavirin (Fig. 1b) [29]. A total of 79 % of the patients had HCV GT-1a infection, 88 % had non-CC IL28B (rs12979860) genotype, and 20 % had cirrhosis. A total of 52 % of the patients had received prior treatment with a protease-inhibitor regimen. Of the total of 440 patients, 55.7 % (245) and 44.3 % (195) were previous treatment relapsers or patients with breakthrough and patients with no response, respectively. Treatment with 12 weeks of the fixed-dose combination of ledipasvir and sofosbuvir, with or without ribavirin, led to SVR12 in 96 % (107/111) or 94 % (102/109) patients, respectively. Treatment with 24 weeks of the fixed-dose combination of ledipasvir and sofosbuvir, with or without ribavirin, led to SVR in 99 % (110/111) or 99 % (108/109) patients, respectively. Of the 11 patients with relapse, 6 had HCV NS5A RAVs at baseline. All these 11 relapsers had HCV NS5A RAVs at the time of relapse. No patients discontinued treatment due to adverse events, of which fatigue, headache and nausea were the most common [29]. In patients with decompensated cirrhosis, ribavirin plays a role in the achievement of SVR [56].

The Japanese study [55] also showed that 12-week treatment with a combination of ledipasvir (90 mg daily) and sofosbuvir (400 mg daily), with or without ribavirin, led to SVR12 in 100 % (87/87) or 100 % (88/88) of treatment-experienced HCV GT-1 patients, respectively.

### Paritaprevir/ritonavir and ombitasvir

A Japanese study (*n* = 73) comprised a randomized, open-label phase 2 trial for HCV GT-1b patients who had not had SVR after treatment with peginterferon and ribavirin with 12 or 24 weeks of the combination of NS3/4A inhibitor paritaprevir (ABT-450)/ritonavir (100/100 mg or 150/100 mg daily) and NS5A inhibitor ombitasvir (ABT-267) (25 mg daily) (Table 1) [57]. SVR 24 weeks after the end of treatment (SVR24) was high (88.9–100 %) regardless of the paritaprevir dose or treatment duration [57]. The most common adverse events were nasopharyngitis (29 %) and headache (14 %).

**Table 1:** Future perspectives of all-oral treatment for HCV genotype 1 in Asian-Pacific Region

Companies (regimen) [references]	Drug targets			Ribavirin	Duration of treatment (weeks)	SVR (%)
	NS3/4A protease	NS5A	NS5B			
Gilead [29, 30, 36, 55]	–	Ledipasvir (90 mg daily)	Sofosbuvir (400 mg daily)	±	12	93–100
Abbvie (2D) [57]	Paritaprevir (150 mg daily)/ritonavir (100 mg daily)	Ombitasvir (25 mg daily)	–	±	12	89–100
Abbvie (3D) [39, 58]	Paritaprevir (150 mg daily)/ritonavir (100 mg daily)	Ombitasvir (25 mg daily)	Dasabuvir (500 mg daily)	±	12	90–100
Bristol-Myers Squibb (2D) [60, 62]	Asunaprevir (200 mg daily)	Daclatasvir (60 mg daily)	–	–	24	81–91
Bristol-Myers Squibb (3D) [64]	Asunaprevir (200 mg daily)	Daclatasvir (60 mg daily)	BMS-791325 (75 or 150 mg daily)	–	12 or 24	89–94
MSD [65]	Grazoprevir (MK-5172) (100 mg daily)	Elbasvir (MK-8742) (50 mg daily)	–	–	12	90–100

Abbvie 2D regimen and Bristol-Myers 2D regimen are only available for HCV genotype-1b infection. Ritonavir is a booster

### Paritaprevir/ritonavir, ombitasvir and dasabuvir

The Turquoise-II study (*n* = 380) consisted of a phase 3 trial for HCV GT-1 patients with Child-Pugh class A cirrhosis with 12 or 24 weeks of the combination of paritaprevir (ABT-450)/ritonavir (150/100 mg daily), ombitasvir (ABT-267) (25 mg daily), NS5B polymerase non-nucleoside inhibitor dasabuvir (ABT-333) (250 mg, twice daily) and ribavirin (Table 1) [39]. Treatment for 12 or 24 weeks led to SVR12 in 91.8 % (191/208) or 95.9 % (165/172) patients, respectively. Drug discontinuations due to adverse events were uncommon (2.1 %).

The PEARL-III and PEARL-IV studies were conducted via a phase 3 trial for HCV GT-1a (*n* = 419) or GT-1b (*n* = 305) patients without cirrhosis, respectively, with a 12-week combination of paritaprevir (ABT-450)/ritonavir (150/100 mg daily), ombitasvir (ABT-267) (25 mg daily), and NS5B polymerase non-nucleoside inhibitor dasabuvir (ABT-333) (250 mg, twice daily), with or without ribavirin (Table 1) [58]. The 12-week treatment, with or without ribavirin, led to SVR12 in 97.0 % (97/100) or 90.2 % (185/205) of the HCV GT-1a patients, respectively. As for the HCV GT-1b patients, the 12-week treatment, with or without ribavirin, led to SVR12 in 99.5 % (209/210) or 99.0 % (207/209), respectively. Drug discontinuations due to adverse events were rare (0.3 %). The most common adverse events were fatigue, headache and nausea.

SAPPHIRE-II studies were conducted by a phase 3 trial for treatment-experienced HCV GT-1 patients without cirrhosis, respectively, with 12-weeks of the combination of paritaprevir (ABT-450)/ritonavir (150/100 mg daily), ombitasvir (ABT-267) (25 mg daily), and dasabuvir (ABT-333) (250 mg, twice daily) with or without ribavirin, (1000 or 1200 mg daily) (Table 1) [40]. The total SVR12 rate was 96.3 % (286/297):95.3 % (82/86) in those with prior relapse, 100 % (65/65) with prior partial response, and 95.2 % (139/146) with prior null response.

### Asunaprevir and daclatasvir

After a phase 2 trial [59], showing that the combination of asunaprevir and daclatasvir was effective only for HCV GT-1b, a Japanese (*n* = 222) randomized, open-label phase 3 trial for HCV GT-1b patients (135 interferon-ineligible/intolerant and 87 non-responder patients) with 24 weeks of the combination of NS3/4A inhibitor asunaprevir (100 mg, twice daily) and NS5A inhibitor daclatasvir (60 mg, once daily) was conducted (Table 1) [60]. SVR24 rates were 87.4 % (118/135) or 80.5 % (70/87) in interferon-ineligible/intolerant or nonresponder patients, respectively. The most common adverse events were nasopharyngitis, ALT and AST elevations, headache, diarrhea and pyrexia. It was also reported that drug-induced immunoallergic hepatitis occurred during the combination therapy with daclatasvir and asunaprevir [61].

The HALLMARK-DUAL study presented a randomized, open-label phase 3 trial for HCV GT-1b patients (205 treatment-naïve, 205 non-responders and 235 ineligible and/or intolerant patients) with the 24-week combination of asunaprevir (100 mg, twice daily) and daclatasvir (60 mg, once daily) (Table 1) [62]. The SVR12 rate was 90 % (182/205), 82 % (168/205), or 82 % (192/235) in treatment-naïve, non-responders, or ineligible and/or intolerant patients, respectively. They [62] detected RAVs at NS5A-L31, NS5A-Y93 and NS3-D168, or a combination of two or more, in 75 of 596 patients. Of these 75 patients, only 29 (39 %) achieved SVR12. Of note, these RAVs were associated with virological failure.

Asunaprevir and daclatasvir are mainly eliminated through liver. Therefore, this combination therapy for 24 weeks is not contraindicated in patients with severe renal impairment, including HCV GT-1 patients with hemodialysis and was highly effective and well tolerated [63].

### Asunaprevir, daclatasvir and BMS-791325

A joint USA/France study (*n* = 64) performed a randomized, open-label phase 2a trial for treatment-naïve HCV GT-1 patients with 12 or 24 weeks of the combination of asunaprevir (200 mg, twice daily), daclatasvir (60 mg, once daily) and BMS-791325 (75 or 150 mg, twice daily) (Table 1) [64]. SVR12 was achieved in 92 % (61/64) of the patients.

### Grazoprevir (MK-5172) and elbasvir (MK-8742)

Another USA/Europian countries study (*n* = 253) consisted of a randomized, open-label phase 2 trial for HCV GT-1 patients with cirrhosis, with 12 or 18 weeks of the combination of HCV NS3/4A inhibitor grazoprevir (MK-5172) (100 mg daily) and NS5A inhibitor elbasvir (MK-8742) (50 mg daily), with or without ribavirin (Table 1) [65]. SVR12 was achieved in 90–97 % of the patients. Less than 1 % of grazoprevir and elbasvir are renally excreted. Therefore, this combination therapy for 12 weeks is not contraindicated in GT-1 patients with stage 4–5 chronic kidney disease and was highly effective and well tolerated [66].

### Future perspectives of chronic HCV GT-1 patients in Asian countries

**Fig. 2:**
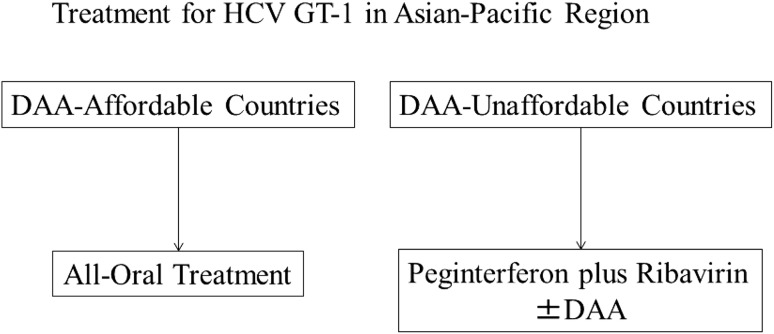
Treatment for HCV genotype-1 in Asian-Pacific region

There are differences between HCV GT-1a and GT-1b according to the several regimens of all-oral treatment for HCV GT-1. HCV subgenotyping and RAVs at HCV NS5A should be analyzed before the commencement of asunaprevir and daclatasvir combination treatment [67]. However, all-oral treatment can be available to cure almost all HCV GT-1 patients including “difficult-to-treat” patients (Fig. 2). Of course, further studies will be needed.

### #2 Consensus Statements and Recommendation on all-oral treatment for HCV GT-1 infection

1. In treatment-naïve patients and previously treated-with peginterferon plus ribavirin patients with chronic HCV GT-1 infection, the following all-oral treatments apply:
  1. For HCV GT-1 patients (treatment-naïve or -experienced with peginterferon plus ribavirin), treatment with daily ledipasvir (90 mg) plus sofosbuvir (400 mg) for 12 weeks is recommended. (A1)
  2. For HCV GT-1b patients (treatment-naïve or -experienced with peginterferon plus ribavirin), other regimens such as paritaprevir/ritonavir, ombitasvir and dasabuvir for 12 weeks or grazoprevir (MK-5172) and elbasvir (MK-8742) for 12 weeks may also be applicable. (A1)
  3. For HCV GT-1b patients (treatment-naïve or -experienced with peginterferon plus ribavirin) with renal impairment, other regimens such as grazoprevir (MK-5172) and elbasvir (MK-8742) for 12 weeks or asunaprevir and daclatasvir for 24 weeks may also be applicable. Before treatment with asunaprevir and daclatasvir, resistant associated variants (RAVs) with target regions should be examined. (A2)
2. In patients previously treated with NS3/4A protease inhibitor and peginterferon plus ribavirin with chronic HCV GT-1 infection, the following all-oral treatments apply:
  1. For HCV GT-1 patients (treatment-naïve or -experienced with peginterferon plus ribavirin), treatment with daily ledipasvir (90 mg) plus sofosbuvir (400 mg) for 12 weeks is recommended. (A1)
3. In patients previously treated with NS5A inhibitor and peginterferon plus ribavirin with chronic HCV GT-1 infection, the following all-oral treatments apply:
  1. For patients with minimal liver disease, treatment should be avoided, until data are available. (C1)
  2. For patients with advanced liver diseases, RAVs with target regions should be examined before any treatments. (C1)

## All-oral treatment for HCV GT-2 and GT-3 infection

### HCV GT-2

In treatment-naive patients with HCV GT-2 infection patients, the all-oral treatment regimen is the combination of sofosbuvir (400 mg per day) with weight-based RBV [1000 mg (<75 kg) to 1200 mg (≥75 kg)] for 12 weeks, which produces high SVR rates (Table 2). This regimen has been evaluated in 4 clinical trials (ELECTRON, FISSION, POSITRON, and VALENCE) [68–71]. The FISSION study randomized patients to receive daily peginterferon and RBV (800 mg) for 24 weeks or sofosbuvir plus daily weight-based RBV [1000 mg (<75 kg) to 1200 mg (≥75 kg)] for 12 weeks [70]. The SVR rate was higher (94 %) in patients who received sofosbuvir plus RBV than in those who received peginterferon and RBV (78 %; 52/67). Across all 3 trials [68, 70, 71], 201 (94 %) of the 214 patients with HCV GT-2 infection achieved SVR with sofosbuvir plus RBV. Among those who did not achieve SVR, sofosbuvir resistance–associated variants (RAVs) were not detected. Based on the results of these trials, both the FDA and EMA approved sofosbuvir and RBV for 12 weeks in all HCV GT-2 treatment-naive patients. An open-label phase III study in Japan confirmed these results [72]. A total of 153 patients with HCV GT-2 infection were enrolled and treated with sofosbuvir plus RBV for 12 weeks. Among them, 11 % had liver cirrhosis and 22 % were over 65 years. Overall, 148 patients (97 %) achieved the SVR. Of the 90 treatment-naive patients, 88 (98 %) achieved SVR, including all the patients with cirrhosis. Of the 63 treatment-experienced patients, 60 (95 %) achieved SVR, including all cirrhosis patients except one (89 %). Thus, the overall SVR rate was 94 % in patients with cirrhosis and those over 65. Although no data were available to support an extension of therapy for treatment-naive patients with HCV GT-2 infection, longer treatment duration may improve SVR in treatment-experienced patients with cirrhosis (60 % after 12 weeks and 78 % after 16 weeks) (Table 3). Therefore, extending treatment from 12 to 16 weeks in HCV GT-2-infected patients with cirrhosis is recommended.

**Table 2:** All-oral treatment for treatment-naïve HCV genotype 2 in the Asian-Pacific Region

Companies (regimen) [references]	Drug targets		Ribavirin	Duration of treatment (weeks)	SVR (%)
	NS5A	NS5B			
Gilead Sciences [68–72]		Sofosbuvir (400 mg daily)	+	12	88–100
Bristol-Myers Squibb [73]	Daclatasvir (60 mg daily)	Sofosbuvir (400 mg daily)	±	24	96
Gilead Sciences [74]	Ledipasvir (90 mg daily)	Sofosbuvir (400 mg daily)	–	12	96
Gilead Sciences [75]	Velpatasvir (GS-5816) (25 or 100 mg)	Sofosbuvir (400 mg daily)	–	12	25 mg: 91 100 mg: 100

**Table 3:** All-oral treatment for treatment-experienced HCV genotype 2 in the Asian-Pacific Region

Companies (regimen) [references]	Drug targets		Ribavirin	Duration of treatment (weeks)	SVR (%)
	NS5A	NS5B			
Gilead Sciences [68, 71]		Sofosbuvir (400 mg daily)	+	12 or 16	12 weeks: 86–95 F4: 60–89 16 weeks: 94 F4: 78

Daclatasvir (60 mg/day) with sofosbuvir (400 mg/day) for 24 weeks was associated with a high SVR rate (96 %) in treatment-naive patients with HCV GT-2 infection [73] (Table 2). Although RBV did not seem to be necessary to achieve SVR, the number of patients was too small to draw any firm conclusions. Ledipasvir (90 mg/day) with sofosbuvir (400 mg/day) for 12 weeks was also associated with high SVR12 rate (96 %) in HCV GT-2 infected patients including treatment-experienced and those with cirrhosis [74] (Table 2). Sofosbuvir in combination with GS-5816 was evaluated in treatment-naive patients with HCV GT-2 infection with high SVR rates [75] (Table 2).

### HCV GT-3

With current anti-HCV agents, HCV GT-3 is the most difficult-to-cure genotype. The VALENCE study assessed the efficacy and safety of sofosbuvir (400 mg per day) plus weight-based RBV [1000 mg (<75 kg) to 1200 mg (≥75 kg)] for 24 weeks [68]. This trial included 250 treatment-naive (42 %) and -experienced (58 %) patients with HCV GT-3 infection. The overall SVR12 rate was 84 % and treatment-naive patients had a higher SVR than -experienced ones (93 vs. 77 %, respectively). These data suggest that higher SVR rates can be achieved with a 24-week sofosbuvir plus RBV therapy than those reported with 12- or 16-week therapy in the FISSION [70] (63 % after 12 weeks), POSITRON [71], (61 % after 12 weeks) and FUSION [71] (30 % after 12 weeks and 62 % after 16 weeks) trials (Tables 4, 5). The SVR rates were comparable between patients with and without cirrhosis (92 and 93 %, respectively). Recently, the phase III ALLY 3 study using daclatasvir (60 mg per day) plus sofosbuvir (400 mg per day) for 12 weeks [76]. The study included 101 treatment-naive patients with HCV genotype 3 infection and showed the SVR rate of 90 %. In treatment-naive patients without cirrhosis (Metavir F0-F3), 97 % achieved SVR, and in treatment-naive patients with cirrhosis (Metavir F4), 58 % achieved SVR.

**Table 4:** All-oral treatment for treatment-naïve HCV genotype 3 in the Asian-Pacific Region

Companies (regimen) [references]	Drug targets		Ribavirin	Duration of treatment (weeks)	SVR (%)
	NS5A	NS5B			
Gilead Sciences [68, 70, 71]		Sofosbuvir (400 mg daily)	+	12 or 24	12 weeks Non-cirrhotic: 61–68 F4: 21–34 24 weeks Non-cirrhotic: 94 F4: 92
Bristol-Myers Squibb [76]	Daclatasvir (60 mg daily)	Sofosbuvir (400 mg daily)	–	12	Non-cirrhotic: 97 F4: 58

**Table 5:** All-oral treatment for treatment-experienced HCV genotype 3 in the Asian-Pacific Region

Companies (regimen) [references]	Drug targets		Ribavirin	Duration of treatment (weeks)	SVR (%)
	NS5A	NS5B			
Gilead Sciences [68, 71]		Sofosbuvir (400 mg daily)	+	16 or 24	16 weeks Non-cirrhotic: 63 F4: 61 24 weeks Non-cirrhotic: 87 F4: 60

### #3 Consensus statements and recommendation on all-oral treatment for HCV GT-2 and GT-3 infection

In chronic HCV GT-2 or GT-3 infection, the following all-oral treatments apply:

1. GT-2
  1. For treatment-naïve HCV GT-2 patients, daily sofosbuvir (400 mg) plus weight-based ribavirin (1000 or 1200 mg in patients <75 kg or ≥75 kg, respectively) for 12 weeks is recommended. (A1)
  2. Therapy can be prolonged to 16 or 24 weeks in cirrhotic patients previously treated with peginterferon plus ribavirin. (A1)
  3. Daily daclatasvir (60 mg) and sofosbuvir (400 mg) for 24 weeks is recommended for treatment-naive patients who cannot tolerate ribavirin. (B1)
  4. Daily ledipasvir (90 mg) and sofosbuvir (400 mg) for 12 weeks is recommended for treatment-naive patients who cannot tolerate ribavirin. (B1)
  5. Daily velpatasvir (25 or 100 mg) and sofosbuvir (400 mg) for 12 weeks is recommended for treatment-naive patients who cannot tolerate ribavirin. (B1)

## Velpatasvir is not yet licensed but will soon be available.

2. GT-3
  1. For treatment-naïve HCV GT-3 patients, treatment with daily sofosbuvir (400 mg) plus weight-based ribavirin (1000 or 1200 mg in patients <75 kg or ≥75 kg, respectively) for 24 weeks is recommended. (A1)
  2. Alternatively, daily daclatasvir (60 mg) and sofosbuvir (400 mg) for 12 weeks (no cirrhosis) (A1) or 24 weeks with or without weight-based RBV (cirrhosis) (B2) is recommended.
  3. For patients previously treated with peginterferon plus ribavirin, daily daclatasvir (60 mg) and sofosbuvir (400 mg) for 12 weeks in those without cirrhosis (A1), and for 24 weeks with weight-based ribavirin for 24 weeks in those with cirrhosis (B2) are recommended.

## PAN-oral therapy for HCV GT-4, GT-5 and GT-6 infection

### Current clinical data with registered agents

For HCV GT-4, GT-5 and GT-6, clinical trials related to the use of interferon-free pan-oral direct acting antiviral agents have been sponsored by AbbVie, Gilead Sciences and Merck (Table 6). Among Egyptians with HCV GT-4 residing in USA, SVR12 was achieved in 79 % (11/14) and 100 % (14/14) of treatment-naive patients treated for 12 weeks and 24 weeks with sofosbuvir plus weight-based RBV [1000 mg (<75 kg) to 1200 mg (≥75 kg)], respectively [77]. Similar results were achieved in a phase II study conducted in Egypt, with SVR12 rates of 84 % (21/25) and 92 % (22/24) in treatment-naive patients treated for 12 weeks and 24 weeks, respectively [78]. In an open-label study of HIV/HCV-co-infected patients (PHOTON-2), 31 treatment-naive patients with HCV GT-4 infection were treated with daily sofosbuvir plus weight-based RBV [1000 mg (<75 kg) to 1200 mg (≥75 kg)] for 24 weeks. In this study, 84 % of patients (26/31) achieved SVR12 [79]. Following the success of ION studies, the SYNERGY trial, an open-label study evaluated a 12-week course of Harvoni (ledipasvir/sofosbuvir) in 21 HCV GT-4-infected patients, of whom 60 % were treatment-naive and 43 % had advanced fibrosis (Metavir F3 or F4). One patient took the first dose and then withdrew consent and SVR12 rate was 95 % by intention-to-treat analysis and 100 % by per-protocol analysis [80]. In PEARL-I, sponsored by AbbVie, treatment-naive patients with HCV GT-4 infection with or without cirrhosis received 12 weeks of the daily fixed-dose combination of Technivie (ombitasvir, paritaprevir and ritonavir) with or without weight-based RBV. SVR12 rates of 100 % (42/42) was achieved in the group receiving RBV and 90.9 % (40/44) in the group not receiving RBV. Adverse effects were generally mild, with headache, asthenia, fatigue, and nausea most commonly reported [81]. In vitro, both ledipasvir and daclactasvir are active against both HCV GT-5 and GT-6. However, clinical data related to the use of these NS5A DAAs are limited. The combination of sofosbuvir and ledipasvir, administered 12 weeks without ribavirin in treatment-naïve and treatment-experienced patients infected with HCV GT-6 yielded SVR rate of 96 % (24/25) [82].

**Table 6:** HCV genotype (GT)-4, GT-5 and GT-6 SVR12 from different regimens

Genotype	Company	Regimen	Non-cirrhotic (LSM < 14.7 kPa)	Cirrhotic (LSM ≥ 14.7 kPa)
			SVR12 (%)/number of treated patients	
GT-4	AbbVie	Ombitasvir plus paritaprevir, 12 weeks	91 % (41/44, treatment-naïve) [81]	No data available
		Ombitasvir plus paritaprevir plus RBV, 12 weeks	100 % (42/42, treatment-naïve or experienced) [81]	No data yet (study is on-going)
	Gilead	Ledipasvir and sofosbuvir (Harvoni™), 12 Weeks	95 % [19/20, treatment-naïve (62 %) or experienced (38 %)] [80]	No data available
		Sofosbuvir plus velpatasvir	100 % (116/116, Sofosbuvir plus velpatasvir 12 weeks) [ASTRAL-1 study] [86]	83 % (75/90, GT1-6, Sofosbuvir plus velpatasvir 12 weeks) [ASTRAL-4 study] [86] 94 % (82/87, GT1-6, Sofosbuvir plus velpatasvir plus ribavirin (RBV) 12 weeks) [ASTRAL-4 study] [86] 86 % (77/90, GT1-6, Sofosbuvir plus velpatasvir 24 weeks) [ASTRAL-4 study] [86]
	MSD	Grazoprevir plus elbasvir	100 % (18/18) [253]	
GT-5	Gilead	Sofosbuvir plus velpatasvir	97 % (34/35, Sofosbuvir plus velpatasvir 12 weeks) [ASTRAL-1 study] [86]	83 % (75/90, GT1-6, Sofosbuvir plus Velpatasvir 12 weeks) [ASTRAL-4 study] [86] 94 % (82/87, GT1-6, Sofosbuvir plus Velpatasvir plus RBV 12 weeks) [ASTRAL-4 study] [86] 86 % (77/90, GT1-6, Sofosbuvir plus Velpatasvir 24 weeks) [ASTRAL-4 study] [86]
GT-6	Gilead	Ledipasvir and sofosbuvir (Harvoni™)	96 % [24/25, treatment-naïve: 23 (92 %); experienced:2 (8 %); Cirrhosis: (8 %)] [80]	
		Sofosbuvir plus velpatasvir	100 % (41/41, Sofosbuvir plus velpatasvir 12 weeks) [ASTRAL-1 study] [86]	83 % (75/90, GT1-6, Sofosbuvir plus velpatasvir 12 weeks) [ASTRAL-4 study] [86] 94 % (82/87, GT1-6, Sofosbuvir plus velpatasvir + RBV 12 weeks) [ASTRAL-4 study] [86] 86 % (77/90, GT1-6, Sofosbuvir plus velpatasvir 24 weeks) [ASTRAL-4 study] [86]

### Emerging clinical study

Future drug combinations will likely exist of two or more DAAs with the aim to achieve (1) pan-genotypic HCV activity, (2) little or no risk for resistance; (3) short duration (≤12 weeks) of treatment, and (4) SVR and definite cure of the disease.

To date, there are limited clinical data to support the use of the combination of 12 weeks of daily sofosbuvir (400 mg) plus simeprevir (150 mg) with or without weight-based RBV [1000 mg (<75 kg) to 1200 mg (≥75 kg)] in HCV GT-4-infected patients. In the open-label phase III RESTORE trial, 107 patients with HCV GT-4 infection, including 35 treatment-naive patients, were treated with simeprevir in combination with peginterferon and ribavirin. In treatment-naive patients, daily simeprevir (150 mg) for 12 weeks in combination with peginterferon and ribavirin for 24–48 weeks (by response-guided therapy) produced SVR in 83 % (29 of 35) [83]. These results are comparable to SVR rates observed with similar regimens in patients with HCV GT-1 infection, suggesting that efficacy of sofosbuvir plus simeprevir for HCV GT-4 infection may be roughly in line with the SVR rates of patients with HCV GT-1 infection shown in the COSMOS trial.

Recently, Merck has developed the combination of grazoprevir, NS3/4A protease inhibitor that has high potency in vitro against HCV GT-1, GT-2, GT-4, GT-5, and GT-6 and elbasvir, NS5A inhibitor active against GT-1, GT-2a, GT-3, GT-4, GT-5, and GT-6, even in the presence of RAVs associated with failure of other NS5A inhibitors, such as daclatasvir and ledipasvir [57, 73]. In the C-EDGE study with oral, once-daily, fixed-dose grazoprevir 100 mg/elbasvir 50 mg for 12 weeks being administered, 18 of 18 (100 %) with HCV GT-4 and 8 (80 %) of 10 with HCV GT-6, achieved SVR [84]. At the time of failure, both patients with HCV GT-6 with virological failure had NS3 resistant associated variants (RAVs), and 1 also had an NS5A RAV [84]. In the pipeline, Gilead Sciences is evaluating the safety and efficacy of an investigational all-oral pan-genotypic regimen containing the nucleotide analog polymerase inhibitor sofosbuvir (SOF) and the investigational NS5A inhibitor velpatasvir (GS-5816) for the treatment of hepatitis C infection across all genotypes. In a phase 2 study, among the 154 previously untreated hepatitis C patients without liver cirrhosis, 9 % had HCV GT-4, a single individual had HCV GT-5 and 6 % had HCV GT-6. About one-third had the favourable IL28B CC gene variant associated with interferon responsiveness. Participants in this open-label study were randomly assigned to receive 400 mg once-daily sofosbuvir plus either 25 mg or 100 mg once-daily GS-5816 for 12 weeks. SVR12 rates for GT-4 SVR12 rates were 100 and 86 %, respectively, in the 25 mg and 100 mg dose groups. The single HCV GT-5 patient and all HCV GT-6 patients in both dose arms were cured [85]. So far, sofosbuvir plus velpatasvir (GS-5816) was well tolerated in over 800 patients with HCV infection evaluated. There was a low incidence of serious adverse effects and few discontinuations due to adverse events. The most frequently reported adverse events (>10 %) were fatigue, headache, nausea and insomnia. The most frequently observed hematologic abnormality was hemoglobin decrease in the RBV-containing treatment groups. Further phase 3 studies, ASTRAL-1,-2, -3 and -4, have recently been completed, with all genotypes evaluated with sofosbuvir 400 mg/velpatasvir (VEL, GS-5816) 100 mg FDC tablet administered orally once daily, with and without RBV. Among the 1035 subjects in the ASTRAL-1, -2, and -3 studies, 21 % had compensated cirrhosis and 28 % had failed prior treatments. In ASTRAL-1, SOF/VEL for 12 weeks had similar adverse events compared with placebo and SVR12 was noted in 100 % (116/116) GT-4, 97 % (34/35) GT-5 and 100 % (41/41) GT-6 [86].

Current availability of interferon-free and ribavirin-free regimens have been shown to be remarkably efficacious and well tolerated. However, the long duration of therapy (12–24 weeks) has raised concerns about adherence and cost [87]. Mirroring from the studies in HCV GT-1, one will anticipate the addition of a third potent direct-acting antiviral drug to the current dual therapy can further reduce the duration of treatment required to achieve sustained viral response in patients with chronic HCV GT-1 infection without cirrhosis. In the proof-of-concept study by Kohli et al., the use of three direct-acting drugs with different mechanisms of action in two therapeutic groups from a mono-infected urban population allowed for a shorter duration of treatment (6 weeks) and resulted in high cure rates and excellent tolerability [88]. Furthermore, viral kinetic modeling suggests that the three-drug regimen of sofosbuvir, ledipasvir, and GS-9451—targeting three different stages of the HCV lifecycle—resulted in enhanced HCV clearance compared with other regimens that target only two stages. Further larger studies will be warranted to confirm these findings and to understand the related specific host or viral factors to better refine our clinical management of these patients.

### #4 Consensus statements and recommendation on all-oral treatment for HCV GT-4 infection

1. Patients without cirrhosis
  1. 12 weeks fixed-dose combination of sofosbuvir (400 mg) and ledipasvir (90 mg) in a single tablet administered once daily without ribavirin. (A1)
  2. 12 weeks fixed-dose combination of sofosbuvir (400 mg) and velpatasvir (VEL, GS-5816) 100 mg FDC tablet administered orally once daily. (A1)
  3. 12 weeks fixed-dose combination of ombitasvir (75 mg), paritaprevir (12.5 mg) and ritonavir (50 mg) in one single tablet (two tablets once daily with food) with daily weight-based ribavirin (1000 or 1200 mg in patients <75 kg or ≥75 kg, respectively. (B1)
2. Patients with compensated cirrhosis
  1. 12 weeks fixed-dose combination of sofosbuvir (400 mg) and velpatasvir (VEL, GS-5816) 100 mg FDC tablet administered orally once daily. (B1)
  2. 24 weeks fixed-dose combination of sofosbuvir (400 mg) and ledipasvir (90 mg) in a single tablet administered once daily without ribavirin. (A1)
  3. 24 weeks fixed-dose combination of ombitasvir (75 mg), paritaprevir (12.5 mg) and ritonavir (50 mg) in one single tablet (two tablets once daily with food) with daily weight-based ribavirin (1000 or 1200 mg in patients <75 kg or ≥75 kg, respectively). (B1)
  4. 12 weeks combination of daily sofosbuvir (400 mg) and daily daclatasvir (60 mg), adding daily weight-based ribavirin (1000 or 1200 mg in patients <75 kg or ≥75 kg, respectively) and extending duration of treatment to 24 weeks without ribavirin. (B2)

## Velpatasvir is not licensed yet but will be available soon.

### #5 Consensus statements and recommendation on all-oral treatment for HCV GT-5 and GT-6 infection

1. Patients without cirrhosis
  1. 12 weeks fixed-dose combination of sofosbuvir (400 mg) and velpatasvir (VEL, GS-5816) 100 mg FDC tablet administered orally once daily. (A1)
  2. 12 weeks fixed-dose combination of sofosbuvir (400 mg) and ledipasvir (90 mg) in a single tablet administered once daily without ribavirin. (B1)
  3. 12 weeks combination of daily sofosbuvir (400 mg) and daily daclatasvir (60 mg). (B1)
2. Patients with compensated cirrhosis
  1. 12 weeks fixed-dose combination of sofosbuvir (400 mg) and velpatasvir (VEL, GS-5816) 100 mg FDC tablet administered orally once daily. (B1)
  2. 12 weeks fixed-dose combination of sofosbuvir (400 mg) and ledipasvir (90 mg) in a single tablet administered once daily with weight-based ribavirin (1000 or 1200 mg in patients <75 kg or ≥75 kg, respectively). (B1)
  3. 24 weeks fixed-dose combination of sofosbuvir (400 mg) and ledipasvir (90 mg) in a single tablet administered once daily without ribavirin. (B1)
  4. 12 weeks combination of daily sofosbuvir (400 mg) and daily daclatasvir (60 mg), adding daily weight-based ribavirin (1000 or 1200 mg in patients <75 or ≥75 kg, respectively). (B1)
  5. 24 weeks combination of daily sofosbuvir (400 mg) and daily daclatasvir (60 mg) without ribavirin. (B1)

## Generic licensing of all-orals in Pakistan

Pakistan has the second highest burden of chronic HCV infection in the world after China. Prevalence of HCV infection is 5 %, with an estimated 8 million people infected with the virus [89]. There are, however, pockets of very high prevalence of up to 24 % of the population [90]. The major HCV genotype in Pakistan is GT-3 (90 % cases), followed by GT-1 (10 % cases). The IL28B genotype is favorable in most cases.

Both standard interferon and peginterferon have been used for treatment of HCV, in combination with ribavirin, with overall SVR rates of 65 % for standard interferon and around 75 % for peginterefon [91]. However, due to the large disease burden, Pakistan was the second country in the world after Egypt to receive sovaldi through the Gilead global access program at the heavily discounted price of 300 USD per month. The drug was formally registered in March 2015 and till now nearly 20,000 patients have been started on treatment. sovaldi is used either in combination with ribavirin for 24 weeks or with ribavirin and peginterferon for 12 weeks. Initial results suggest SVR rates that are similar to those reported for HCV GT-3, of around 85 %.

A sizeable population of difficult to treat HCV GT-3 patients with prior treatment exposure as well as cirrhosis is emerging, particularly in the tertiary care centers. For these patients, additional DAAs are urgently needed.

## New DAA in Egypt: the licensing scenario

It seems that, finally, the nightmare of HCV in Egypt will end, as by April 2015, 2 DAA brands, Sofosbuvir, and Semiprevir. as well as 2 generics for Sofosbuvir have been registered and are available in Egypt. Early in 2014, the Egyptian government represented by its Ministry of Health (MOH) and the National Committee for Control of Viral Hepatitis (NCCVH), successfully signed a memorandum of understanding (MoU) with Gilead according to which Gilead accepted to supply its DAA brand, Sofosbuvir, at only 1 % of its price in the USA. This price was close to 300 USD/28 cap box. By March 2015, the Ministry of Health signed a similar MoU with Janssen to supply its brand Simeprevir at a price of 250 USD/box. Simeprevir has recently been added to the national treatment program.

- Other approved treatment options for HCV GT-4 as, LEDIPASVIR/SOFOSBUVIR “Harvoni”, Daklatasvir and Viekirax [92] will soon be available.
- The National Committee for Control of Viral Hepatitis (NCCVH), finalized the regulations for using these new brands. This current phase of the national program to treat HCV in Egypt was preceded by a state announcement that has been spread in all Egyptian media, starting from September 2014, requesting all Egyptian patients known to have HCV with advanced liver disease to register on the website, www.nccvh.org.e.g.
- Electronic replies to patients were used to fix individual appointment dates and referred to a center where each patient has to be assessed for legibility to use the new treatment protocols.
- By September 18 2014, 180,000 Egyptians have registered on the website, with 10 % of those registered living outside Egypt. By March 2015, 850,000 patients in total were already registered and processed through the website.
- Thirty-four governmental centers are currently participating in the project. The number is increasing monthly to cover all governorates.
- The first doses were distributed starting October 20 2014 and 25,000 patients had been treated by March 2015. It is planned that more than 250,000 patients yearly would receive the new treatment protocols. The same rules will be applied for all patients regardless of the source of payment, and there will be no place for patients’ preferences in deciding the treatment regimen.
- Treatment in its first phase included only cases with F2, F3, F4, or compensated cirrhosis, excluding cases with decompensation or with HCC patients except after successful radical curative intervention (4 months after resection or successful local ablation) evident by triphasic CT.
- The presence of large risky esophageal varices, required prophylactic management before being treatment-eligible.
- Age limits for treatment eligibility fixed to be above 18 years and below 70 years for all patients while body mass index (BMI) will be accepted up to 35.
- For special population groups; priority for treatment will be offered for post-liver transplantation, post-kidney transplantation patients and combined HCV/HBV infection regardless of the fibrosis stage. Other groups such as pediatric age groups and kidney disease patients will be reviewed following the availability of sufficient data. Patients with documented extra-hepatic manifestations will be prioritized for treatment according to the same guidelines.
- Treatment-experienced patients should only start 6 months after cessation of the previous therapy.
- No differentiation in treatment priority will be established based on the previous treatment experience.
- Sofosbuvir (nucleotide polymerase inhibitor) was introduced in the national treatment program through 2 treatment options:

1. Triple Regimen including peginterferon (α2A and α2B): Sofosbuvir plus peginterferon and ribavirin for 12 weeks
2. Dual Regimen: Sofosbuvir plus RBV for 24 weeks

Treatment was designed for those with Fibrosis score F2, F3, F4 and compensated cirrhotics. Recent published reports support this recommended protocol [93].

- The increasing number of reports that treatment with the new DAAs are well tolerated and efficacious in patients with decompensated cirrhosis and, more importantly, markers of hepatic and synthetic function improved during the short-term follow-up [94, 95], will be reviewed. The inclusion of these cases in the national program will be considered during the next phases of the national treatment program.
- In the new era of the DAAs, in addition to increased treatment efficacy with SVR more than 90 %, by increasing cases detection and reducing new infections, according to a mathematical modeling, the government strategy is to achieve <2 % prevalence by 2025 and >90 % drop in prevalence by 2030 (near-total HCV elimination) [96].

**Results of the national program** The initial report of the national program was published in June 2015. The report was an interim analysis of efficacy in both treatment groups (dual and triple therapy). The study included 458 patients received dual regimen and 349 received triple regimen. Both groups were evaluated for treatment results at week 12 which is considered the end of treatment for the triple regimen and midpoint of treatment in the dual regimen.

### Results

Detailed results of the analysis are shown in Table 7. The overall treatment response at week 12 in both groups was 98.8 % (798/807), ETR in the triple regimen group was 99.1 % (346/349) and in the dual regimen group was 98.3 % (452/458) [97].

**Table 7:** Patients characteristics according to treatment response

	Treatment responders (*n* = 798)	Treatment non-responders (*n* = 9)	*p* value
Gender			
Male	548	5	0.47
Female	250	4	
Age (mean ± SD)	53 ± 8	57 ± 6	0.16
Treatment status			
Naïve	540	3	0.07*
Experienced	258	6	
Baseline laboratory values (mean ± SD)			
Hemoglobin	13.4 ± 1.7	13.3 ± 1.6	0.82
WBCs	5.8 ± 4	5.1 ± 2	0.66
Platelets	129 ± 66	133 ± 92	0.88
Albumin	3.8 ± 0.7	3.8 ± 0.8	0.87
Viremia (log_10_)	5.5 ± 1	5.5 ± 0.7	0.98
Fibrosis assessment			
FIB-4 median (IQR)	4.34 (4.2)	6.6 (4.5)	0.23
Fibrosis grading (*n* = 257) F2/F3/F4	2/171/84	1/2/0	<0.01*
Child score			
Non cirrhotic/A/B	535/226/37	3/4/2	0.08/0.46
Regimen type			
Dual	452	6	0.79
Triple (Roch/MSD)	346(198/148)	3(2/1)	0.36
Abdominal U/S			
Liver cirrhosis	482	5	0.96
Ascites	17	0	0.47
Sponsorship			
Governmental	715	5	

* Significant* p* < 0.05

### #6 Consensus statements and recommendation on generic licensing of all-orals in Egypt

1. In the new era of the DAAs, with the high clearance rate of the HCV and minimal side effects, the dream of global eradication of the virus seems feasible. (A1)
2. The major global obstacle in using these new DDAs is the outstanding very high prices of these drugs exceeding the capabilities of health care systems even in rich developed countries, and consequently the scenario will be harder both in poor as well as in developing countries with low national incomes and financial resources. (B1)
3. In developing countries like Egypt having a high prevalence of HCV infection, in parallel with relatively low health care budgets and resources, the only feasible strategy to eradicate the virus is to establish deals with these new DAAs promising drug-producing companies to supply the drugs at a cheap affordable price for both the government and individuals. (B1)

## Generic licensing of all-orals in Indonesia

In Indonesia, chronic hepatitis C infection is dominated by GT-1b. Therefore, the standard of care (SOC) is the combination of peginterferon-alpha 2a or 2b with ribavirin [98]. Based on a multicenter prospective open-label trial in Indonesia using conventional interferon therapy with ribavirin, it was shown that SVR was 78.3 % in patients with HCV GT-1 and GT-4, and 100 % in non-GT-1 and GT-4 [99], whereas in a recent publication based on IL-28B distribution, the 73.5 % SVR was achieved in chronic HCVGT-1 patients with 75.8 % SVR in CC genotype using peginterferon-alpha 2a and ribavirin therapy [100].

### DAA in Indonesia

The only available DAA in Indonesia at this moment is boceprevir which should be used together with peginterferon and ribavirin. Sofosbuvir will soon be available in Indonesia with generic licensing. Since most Indonesian patients are dominated by HCV GT-1, the use of peginterferon will still be the cornerstone of standard therapy. However, the potential for an upcoming new combination of DAA agents with the possibility of generic licensing will give a new standard of care in Indonesia.

### #7 Consensus statements and recommendation on generic licensing of all-orals in Indonesia

1. In Indonesia, chronic hepatitis C infection can be treated with the standard therapy which consists of peginterferon and ribavirin. (B1)
2. Boceprevir might be used in addition to standard therapy if only there is no or partial response to the standard therapy. (B2)
3. Sofosbuvir might be used in combination with ribavirin in HCV GT-2 and GT-3 patients. (B1)
4. An upcoming new combination of DAA agents might be used or replace the use of peginterferon as a therapy for chronic HCV infection in all genotypes. (A1)

## HCV infection in patients with hepatic decompensation, liver transplant candidates and recipients

HCV reinfection after liver transplant is universal and inevitable, and can be the key factor associated with premature graft loss. Treatment of HCV infection is crucial, as successful treatment has the potential to alter the outcomes of decompensated liver disease, liver transplant candidates and in patients with HCV recurrence after transplantation. Previously, interferon-based therapies have not been used in decompensated liver disease due to the high risk of infection and further hepatic decompensation [101]. The outcomes of interferon-based therapy after liver transplantation have been relatively poor compared to interferon-based therapies in the non-transplant situation [102]. The advent of interferon-free therapies enables successful treatment in liver transplant candidates, patients with hepatic decompensation (proceeding or not to liver transplantation) and patients in the post-transplant situation.

- *The liver transplant candidate with HCC and portal hypertension but no liver decompensation*. There has only been one published study of interferon-free antiviral therapy in this situation. Curry et al. reported on using sofosbuvir and ribavarin in 61 patients awaiting liver transplantation for HCC as the primary indication [38]. The majority of patients had HCV GT-1. Patients had CPS < 7. Nine patients relapsed, did not respond or had viral breakthrough whilst 47 underwent liver transplantation. Of these, 3 had HCV detected at transplant and all 3 relapsed post-transplant. Forty-three patients were PCR negative at transplant and 10 relapsed post-transplant. The authors claimed that 24/25 patients who had the virus not detected for >4 weeks and underwent transplant had no recurrence. Thus, it seems that 30/61 patents (49 %) on an intention-to-treat basis had no HCV recurrence in the allograft. This paper has established the proof of principle that prolonged viral suppression for >4 weeks pre-transplant with sofosbuvir-based therapy can prevent reinfection of the liver allograft in at least 50 % of patients.
- *Decompensated Cirrhosis*. There are now several reports (all still in abstract form) using sofosbuvir-based interferon-free antiviral therapy (AVT) in this situation [103–107]. These studies mainly on HCV GT-1 report SVR rates for sofosbuvir and ledipsavir of 80–90 % in Child-Pugh Score C patients but this may be as low as 70 % [103–107]. In these studies, an improvement of >2 in MELD scores was seen in 40–50 % of patients but worsened in about 5–10 %. In an open label study of simeprevir and sofosbuvir in HCV GT-1 patients, a similar SVR (approximately 80 %) was seen. However, simeprevir drug levels may have a significantly increased area under the curve in decompensated diseases and is generally not recommended. In one study of HCV GT-3 patients, sofosbuvir plus daclatasvir resulted in a SVR of 70 versus 60 % versus sofosbuvir plus ledipasvir [105]. It remains unclear whether ribavarin results in an increased SVR in these patients using such regimes. It seems that HCV GT-2 patients may have similar response rates using sofosbuvir and ribavirin.
- *Treating post-liver transplant HCV recurrence*. A study of treatment of mild hepatitis C allograft infection with an AbbVie 3D regimen (ritonavir boosted paritaprevir, ombitasvir and dabuvir) in HCV GT-1 infection led to a 90 % SVR rate [37]. Similar SVR rates have been seen in sofosbuvir and ledipasvir therapy including some patients with more advanced disease [108–112]. Studies with sofosbuvir-based therapies in the post-transplant population in very severe liver disease such as cholestatic hepatitis C have led to 60–70 % SVR rates, but in such studies there was a mortality of approximately 13 % due to advanced disease [113, 114]. Ribavirin should be added on the sofosbuvir-based therapies and the duration of the regimens should be 24 weeks in patients with decompensated liver disease or in HCV GT-3 patients of post-transplant patients [56].

In conclusion there seem to be limitations to the outcomes from antiviral therapy particularly in decompensated liver disease. Current data suggest that interferon-free AVT will prevent HCV allograft reinfection in a significant number of patients. However, many patients with severe portal hypertension and ascites may continue to have hepatic decompensation and severe portal hypertension despite achieving SVR, and thus will still require liver transplantation. It is not clear what parameters exist that are determining successful reversible portal hypertension or not. Furthermore, sofosbuvir-based therapies have not been studied extensively in patients with renal dysfunction which is not an uncommon problem in advanced liver disease. (Sofosbuvir is currently contraindicated in patients with creatinine clearance rates of less than 30 mL/min.) Thus, sofosbuvir-based regimes may not be a viable option in patients with very high MELD scores who have overt or incipient renal failure.

### #8 Consensus statements and recommendation on decompensated liver disease

1. All the patients with decompensated liver disease can be treated with interferon-free-based therapies. Emerging data suggest that sofosbuvir-based treatment plus or minus ledipasvir (HCV GT-1) or daclastasvir (HCV GT-3) may be effective in this setting. Patients should be treated for a period of 12–24 weeks. Anticipated SVR rates at between 70 and 90 %. (B1)

### #9 Consensus statements and recommendation on awaiting liver transplant

1. All patients awaiting liver transplantation can be treated as above with interferon-free antiviral therapy (sofosbuvir-based treatment plus or minus ledipasvir (HCV GT-1) or daclastasvir (HCV GT-3) for a minimum of 3 months before liver transplantation to prevent allograft recurrence. If viral clearance persists for >4 weeks even on-treatment, allograft re-infection is very likely to be prevented.(A1). It remains unclear how many patients improve MELD and CTP scores that allow removal from waiting lists. Thus, interferon-free therapy may best be introduced post-transplant rather than pre-transplant in patients with very high MELD scores. (C1)

### #10 Consensus statements and recommendation on following liver transplant

1. Following liver transplantation recurrent HCV infection should be treated with interferon-free based therapies for 3 months. It is reasonable to commence therapy between 1 and 3 months post-transplant. These regimens may include sofosbuvir and ledipasvir or daclatasvir and the Abbvie 3D regime (ritonavir-boosted paritaprevir, ombitasvir and dabuvir) for mild disease. If the latter is used, dose adjustments (significant reductions) of cyclopsorin and tacrolimus are required. For more severe disease, evidence exists for sofosbuvir-based therapy in the combination of ledipasvir (GT-1) or daclatasvir (GT-3). (B1)
2. With the introduction of universal antiviral treatment post-transplant, the issues of immunosuppression should not be different in this population than in other liver transplant populations, although immunosuppressive drug doses may need adjustment depending on the regime used. (C2)

## SVR for renal failure and co-infection with HBV/HIV

### SVR for HCV infection in patients with renal failure

Comorbidities of HCV infection and chronic kidney disease (CKD) might present in two ways: HCV infection during maintenance dialysis and HCV-associated kidney disease. These disorders can occur both in native kidneys and in renal allografts. Therefore, all patients with kidney diseases should be evaluated for possible underlying HCV infection. It is suggested that HCV-infected patients be tested at least annually for proteinuria and hematuria.

The prevalence of HCV increases with the time that CKD patients are on dialysis which suggests that nosocomial transmission may be the route of infection in previously uninfected dialysis patients [115]. Sensitive quantitative RT-PCR tests for HCV should be administered to hemodialysis patients with unexplained abnormal aminotransferase(s) levels. Patients with end-stage renal disease (ESRD) have lower serum ALT levels than the general population; thus, some studies suggest that the optimized cut-off ALT level be approximately 0.4–0.45 times the upper limit of normal for HCV-infected patients [1, 2, 116, 117]. Cirrhosis, Asian race and history of alcohol abuse are associated with the highest risks for the development of HCC among dialysis patients with HCV infection [118]. Even after adjusting for concurrent co-morbidities, HCV infection is associated with higher risk of liver-related mortality in these subgroups of HCV-infected patients. Furthermore, HCV infection decreases the health-related quality of life in dialysis patients.

Currently, DAA-based therapies offer the best outcomes in patients with chronic kidney disease (CKD) who have mild to moderate renal impairment, i.e., creatinine clearance (CCr) from 30 mL/min to 80 mL/min. The standard dose of sofosbuvir, fixed-dose combination of sofosbuvir/ledipasvir, simeprevir, fixed-dose combination of paritaprevir/ritonavir/ombitasvir plus dasabuvir have demonstrated good efficacy in CKD. Simeprevir was also used safely at standard dose for patients with severe renal impairment, i.e., CCr < 30 mL/min.

In many countries in Asia, the access to new DAAs is limited and variable, and thus the combination peginterferon-alpha and ribavirin may still be the standard of care available for HCV infection in many CKD patients with normal, mild, moderate, or severe decrease in the glomerular filtration rate (GFR), and even in those with kidney failure. For interferon-based treatment regimens, please refer to the 2012 edition of this clinical practice guidelines for further verification [1].

### #11 Consensus statements and recommendation on HCV and CKD

1. HCV-infected patients should be screened for proteinuria and hematuria at least annually to detect early HCV-associated kidney disease. (B2)
2. Maintenance hemodialysis (CKD stage 5D) confers a significant risk of nosocomial infection. Therefore, standard precautions for prevention of nosocomial infections must be rigorously observed. (A1)
3. Patients on hemodialysis should be screened with serological tests and RT-PCR at first hemodialysis or when transferring from another hemodialysis unit. (A1)
4. Maintenance hemodialysis patients and kidney transplant candidates should be tested for anti-HCV antibodies every 6–12 months, and RT-PCR should be performed for patients with unexplained elevated aminotransferase(s). (B2)
5. In dialysis patients with chronic HCV infection, liver biopsy is not mandatory, but may be recommended when the results would influence treatment decisions and when progression of the liver disease needs to be assessed. (B2)
6. Peginterferon with or without ribavirin is recommended for HCV-infected CKD patients with normal or mild decrease in GFR [creatinine clearance (CCr) ≈ 60 mL/min]. (B2)
7. The standard dose of sofosbuvir, fixed-dose combination of sofosbuvir/ledipasvir, simeprevir, fixed-dose combination of paritaprevir/ritonavir/ombitasvir plus dasabuvir may be used in the treatment or re-treatment of patients with mild to moderate renal impairment, i.e., CCr 30–80 mL/min. The use of these agents for patients with severe renal impairment, i.e., CCr < 30 mL/min or with ESRD has not been tested. (B2)
8. Regular serological screening of dialysis staff is highly recommended. (B2)

### SVR for HCV co-infection with HIV

Hepatitis C has a limited impact on HIV disease progression. Conversely, HIV alters the natural history of hepatitis C in several important areas [2]. A rapid progression of liver fibrosis and increased mortality after decompensation has been observed in HCV/HIV co-infected patients [119–121].

HCV/HIV co-infected individuals should be offered treatment or re-treatment like any other individual without HIV infection, regardless of their stage of fibrosis at diagnosis [122–125].

Second generation DAA-based therapies have demonstrated high efficacy and safety in treatment-naïve, treatment experienced and cirrhotic HCV patients co-infected with HIV. Please refer to the “PAN-oral therapy for HCV GT-4, GT-5 and GT-6 infection” section for this guideline’s recommended DAA-based regimens for HCV infection. However, caution should be exercised when using such agents due to known drug–drug interactions with antiretroviral agents. A close collaboration with HIV specialist is recommended when treating HCV/HIV co-infected individuals.

While current western guidelines do not favor peginterferon/ribavirin-based therapies any more, in Asia, many of the second-generation DDAs are not yet available and thus combination peginterferon and ribavirin is still the standard of care. Data from previous studies have indicated that SVR achieved with this regimen reduces liver-related complications and mortality in HCV/HIV co-infected patients [126, 127]. Predictors of treatment response with peginterferon/ribavirin therapy are factors largely related to HCV: rapid virologic response (RVR), HCV genotype, HCV viral load, IL28B gene variation, and liver disease stage, however, the SVR rates in HIV/HCV co-infected patients are 15–20 % lower than those in patients with HCV mono-infection. Likewise, rates of hepatic decompensation during peginterferon/ribavirin treatment are considerably higher in co-infected patients than in HCV mono-infected patients, especially among cirrhotics [2, 128–133]. For interferon-based treatment regimens, please refer to the previous report for further verification [1].

### #12 Consensus statements and recommendation on HCV and HIV co-infection

1. Routine screening for HIV is recommended in patients with hepatitis C following exposure risk assessment and pretest counselling. (A1)
2. HIV/HCV co-infected patients with advanced HIV disease (CD4 count < 100/μL) should receive highly active anti-retroviral therapy (HAART). HCV treatment should be delayed until immune function is improved, preferably until a CD4 count >200/μL is achieved. (A1)
3. Antiretroviral therapy-naïve HIV/HCV co-infected patients with a CD4 count of 100–350/μL should commence HAART prior to HCV treatment. (A1)
4. HIV/HCV co-infected patients with a CD4 count >350/μL should be considered for HCV treatment and do not require HAART. (A1)
5. HCV/HIV co-infected individuals should be offered HCV treatment or re-treatment like any other individual without HIV infection, regardless of the stage of fibrosis at diagnosis. (B1)
6. In regions in Asia where DAAs are not yet available/accessible, peginterferon and ribavirin combination therapy for 48 weeks is still the recommended HCV treatment; weight-based ribavirin dosing should be considered for HCV genotype 1 patients. (A1)
7. Drug–drug interactions of ribavirin- and DAA-based HCV medications with antiretroviral agents may induce adverse reactions and unwanted toxicities. (A1)
8. Ledipasvir should not be used in patients with CCr < 60 mL/min when co-administered with tenofovir-containing regimens. (B2)
9. Baseline and regular on-treatment evaluation of renal function is recommended when medications known to increase tenofovir levels are used. (B2)
10. Tipranivir, cobicistat and elvitegravir (pending more current data) should not be used with fixed-dose sofosbuvir/ledipasvir. (B2) Tipranivir should not be used with sofosbuvir. (B2)
11. Fixed-dose combination paritaprevir/ritonavir/ombitasvir plus dasabuvir should not be co-administered with ritonavir-boosted lopinavir, efaverinz, rilpivirine, and darunavir. (B2)
12. When used for boosting HIV protease inhibitor (PI), adjustments in the dose of ritonavir may be necessary if used with paritaprevir/ritonavir/ombitasvir plus dasabuvir. (B2)
13. Simeprevir should not be used with efaverinz, etraverine, nevirapine, cobicistat, or any HIV protease inhibitors. (B2)
14. Regimens containing telaprevir or boceprevir and monotherapy with peginterferon, ribavirin or a DAA are not recommended. (A1)

### SVR for HCV co-infection with HBV

If HCV is determined to be replicating and is the dominant driver of liver inflammation in HCV/HBV co-infection, co-infected patients should be treated with similar regimens like those with HCV monoifection. When serum HBV DNA levels are elevated at any time before, during and after HCV treatment, nucleos(t)ide analogues may be added to current HCV therapy. Peginterferon may be an option. Please refer to the recommendations in the 2012 guidelines [1]. Similar SVR rates are achieved with HCV genotype-guided peginterferon/ribavirin therapy in HCV mono-infected versus HCV/HBV co-infected patients [134]. HBsAg seroclearance is also observed in co-infected patients treated with peginterferon/ribavirin [135–137]. Some of the newly-approved DAAs for HCV treatment have demonstrable drug–drug interactions with nucleos(t)ide analogues which may limit or preclude their combined use in the HCV/HBV co-infected patients. In patients who achieved SVR, long-term follow-up and monitoring for relapse of HBV infection is recommended [124].

### #13 Consensus statements and recommendation on HCV and HBV co-infection

1. Routine screening for HBsAg is recommended in patients with chronic HCV infection, especially in IVDUs or other high-risk populations. (A1)
2. Routine testing for serum HBV DNA is not recommended in HBsAg-negative patients with chronic HCV infection. (B1)
3. HCC screening tests, including liver ultrasonography and tests for AFP levels, are also required for co-infected patients. (B1)
4. HBV and HCV co-infected patients may be selected for antiviral treatment by the same criteria as those used for patients with mono-infection. (B2)
5. Determine which virus is dominant in patients with dual infection before commencing treatment. When HCV is the dominant replicating virus, treatment regimens are similar to those with HCV mono-infection. (B1)
6. At any time the serum HBV DNA is elevated, appropriate anti-HBV treatment must be started. (A1)
7. Baseline and regular on-treatment evaluation of renal function is recommended when tenofovir is used concurrently with DAAs and other anti-HCV medications known to increase tenofovir levels. (A1)
8. Co-administration of ledipasvir with tenofovir is not recommended in patients with CCr < 60 mL/min. (B2)
9. Simeprevir and fixed-dose paritaprevir/ritonavir/ombitasvir plus dasabuvir do not have clinically significant interactions with lamivudine and tenofovir. (B2)
10. HBV vaccination should be offered for hepatitis C patients who are HBsAg negative. (A1)

In conclusion, we have reviewed the recent advances of interferon-free therapies for the patients with chronic HCV infection in Asian-Pacific countries. A summary is shown in Table 8 and Supplementary Table 2. In the near future, HCV treatment will be further evolved to achieve much higher SVR rates and much shorter treatment duration.

**Table 8:** Interferon-free treatment for patients infected with HCV various genotypes (GTs)

Grading	TN/TE-*PR*		TE-*NS3/4A inhibitor*		TE-*NS5A inhibitor*	
	Non-cirrhosis/cirrhosis		Non-cirrhosis/cirrhosis		Non-cirrhosis	Cirrhosis
(A) GT-1						
A1	SOF/LDV for 12 weeks		SOF/LDV for 12 weeks			
	(GT-1b) PrOD for 12 weeks					
	(GT-1b) GZR/EBR for 12 weeks					
A2	(GT-1b and RAV Check) ASN/DCV for 24 weeks					
C1					Wait	RAV check

Grading	TN		TE-*PR*	
	Non-cirrhosis	Cirrhosis	Non-cirrhosis	Cirrhosis
(B) GT-2				
A1	SOF/RBV for 12 weeks	SOF/RBV for 12 weeks	SOF/RBV for 12 weeks	SOF/RBV for 12 weeks
A1				SOF/RBV for 16-24 weeks
B1 (for RBV-intolerant)	SOF/DCV for 24 weeks SOF/LDV for 12 weeks SOF/VEL for 12 weeks	SOF/DCV for 24 weeks SOF/LDV for 12 weeks SOF/VEL for 12 weeks	SOF/DCV for 24 weeks SOF/LDV for 12 weeks SOF/VEL for 12 weeks	SOF/DCV for 24 weeks SOF/LDV for 12 weeks SOF/VEL for 12 weeks

Grading	TN		TE-*PR*	
	Non-cirrhosis	Cirrhosis	Non-cirrhosis	Cirrhosis
(C) GT-3				
A1	SOF/RBV for 24 weeks	SOF/RBV for 24 weeks	SOF/RBV for 24 weeks	SOF/RBV for 24 weeks
A2	SOF/DCV for 12 weeks	SOF/DCV ± RBV for 24 weeks	SOF/DCV for 12 weeks	
B2		SOF/RBV for 16 weeks		SOF/DCV/RBV for 24 weeks

Grading	Non-cirrhosis	Cirrhosis
(D) GT-4		
A1	SOF/LDV for 12 weeks SOF/VEL for 12 weeks	SOF/LDV for 24 weeks
B1	PrO/RBV for 12 weeks	PrO/RBV for 24 weeks SOF/VEL for 24 weeks
B2		SOF/DCV/RBV for 12 weeks SOF/DCV for 24 weeks
(E) GT-5/GT-6		
A1	SOF/VEL for 12 weeks	
B1	SOF/LDV for 12 weeks	SOF/DCV for 12 or 24 weeks
B1	SOF/LDV for 12 weeks	SOF/LDV for 12 or 24 weeks
B1		SOF/VEL for 12 weeks

*TN* treatment-naïve;* TE* treatment-experienced;* PR* peginterferon plus ribavirin;* SOF/LDV* sofosbuvir/ledipasvir;* PrOD* paritaprevir/ritonavir/ombitasvir/dasabuvir;* GZR/* *EBR* grazoprevir/elbasvir;* ASN/DCV*, asunaprevir/daclatasvir;* RAV* resistant associated variant

*Vel*,velpatasvir (will soon be available)

## Electronic supplementary material

Below is the link to the electronic supplementary material.

Supplementary material 1 (DOCX 31 kb)

## References

[1] Omata M, Kanda T, Yu ML, Yokosuka O, Lim SG, Jafri W, Tateishi R, Hamid SS, Chuang WL, Chutaputti A, Wei L, Sollano J, Sarin SK, Kao JH, McCaughan GW (2012). APASL consensus statements and management algorithms for hepatitis C virus infection. Hepatol Int.

[2] Asian-Pacific Association for the Study of the Liver (APASL) Hepatitis C Working Party, McCaughan GW, Omata M, Amarapurkar D, Bowden S, Chow WC, Chutaputti A, Dore G, Gane E, Guan R, Hamid SS, Hardikar W, Hui CK, Jafri W, Jia JD, Lai MY, Wei L, Leung N, Piratvisuth T, Sarin S, Sollano J, Tateishi R (2007). Asian-Pacific Association for the Study of the Liver consensus statements on the diagnosis, management and treatment of hepatitis C virus infection. J Gastroenterol Hepatol.

[3] Kanda T, Imazeki F, Yokosuka O (2010). New antiviral therapies for chronic hepatitis C. Hepatol Int.

[4] Zeuzem S, Hultcrantz R, Bourliere M, Goeser T, Marcellin P, Sanchez-Tapias J (2004). Peginterferon alfa-2b plus ribavirin for treatment of chronic hepatitis C in previously untreated patients infected with HCV genotypes 2 or 3. J Hepatol.

[5] Mangia A, Santoro R, Minerva N, Ricci GL, Carretta V, Persico M (2005). Peginterferon alfa-2b and ribavirin for 12 vs. 24 weeks in HCV genotype 2 or 3. N Engl J Med.

[6] Shiffman ML, Suter F, Bacon BR, Nelson D, Harley H, Sola R (2007). Peginterferon alfa-2a and ribavirin for 16 or 24 weeks in HCV genotype 2 or 3. N Engl J Med.

[7] Dalgard O, Bjoro K, Ring-Larsen H, Bjornsson E, Holberg-Petersen M, Skovlund E (2008). Pegylated interferon alfa and ribavirin for 14 versus 24 weeks in patients with hepatitis C virus genotype 2 or 3 and rapid virological response. Hepatology.

[8] Lagging M, Langeland N, Pedersen C, Farkkila M, Buhl MR, Morch K (2008). Randomized comparison of 12 or 24 weeks of peginterferon alpha-2a and ribavirin in chronic hepatitis C virus genotype 2/3 infection. Hepatology.

[9] Yu ML, Dai CY, Huang JF, Hou NJ, Lee LP, Hsieh MY, Chiu CF (2007). A randomised study of peginterferon and ribavirin for 16 versus 24 weeks in patients with genotype 2 chronic hepatitis C. Gut.

[10] Jensen DM, Morgan TR, Marcellin P, Pockros PJ, Reddy KR, Hadziyannis SJ (2006). Early identification of HCV genotype 1 patients responding to 24 weeks peginterferon alpha-2a (40 kd)/ribavirin therapy. Hepatology.

[11] Yu ML, Dai CY, Huang JF, Chiu CF, Yang YH, Hou NJ, Lee LP (2008). Rapid virological response and treatment duration for chronic hepatitis C genotype 1 patients: a randomized trial. Hepatology.

[12] Zeuzem S, Buti M, Ferenci P, Sperl J, Horsmans Y, Cianciara J (2006). Efficacy of 24 weeks treatment with peginterferon alfa-2b plus ribavirin in patients with chronic hepatitis C infected with genotype 1 and low pretreatment viremia. J Hepatol.

[13] Liu CH, Liu CJ, Lin CL, Liang CC, Hsu SJ, Yang SS (2008). Pegylated interferon-alpha-2a plus ribavirin for treatment-naïve Asian patients with hepatitis C virus genotype 1 infection: a multicenter, randomized controlled trial. Clin Infect Dis.

[14] Berg T, Weich V, Teuber G, Klinker H, Möller B, Rasenack J (2009). Individualized treatment strategy according to early viral kinetics in hepatitis C virus type 1-infected patients. Hepatology.

[15] Pearlman BL, Ehleben C (2014). Hepatitis C genotype 1 virus with low viral load and rapid virologic response to peginterferon/ribavirin obviates a protease inhibitor. Hepatology.

[16] Ge D, Fellay J, Thompson AJ, Simon JS, Shianna KV, Urban TJ, Heinzen EL (2009). Genetic variation in IL28B predicts hepatitis C treatment-induced viral clearance. Nature.

[17] Tanaka Y, Nishida N, Sugiyama M, Kurosaki M, Matsuura K, Sakamoto N, Nakagawa M (2009). Genome-wide association of IL28B with response to pegylated interferon-alpha and ribavirin therapy for chronic hepatitis C. Nat Genet.

[18] Suppiah V, Moldovan M, Ahlenstiel G, Berg T, Weltman M, Abate ML (2009). IL28B is associated with response to chronic hepatitis C interferon-alpha and ribavirin therapy. Nat Genet.

[19] Huang CF, Huang JF, Yang JF, Hsieh MY, Lin ZY, Chen SC, Wang LY (2012). Interleukin-28B genetic variants in identification of hepatitis C virus genotype 1 patients responding to 24 weeks peginterferon/ribavirin. J Hepatol.

[20] Thompson AJ, Muir AJ, Sulkowski MS, Ge D, Fellay J, Shianna KV (2010). Interleukin-28B polymorphism improves viral kinetics and is the strongest pretreatment predictor of sustained virologic response in genotype 1 hepatitis C virus. Gastroenterology.

[21] Huang CF, Yeh ML, Huang JF, Yang JF, Hsieh MY, Lin ZY (2012). Host interleukin-28B genetic variants versus viral kinetics in determining responses to standard-of-care for Asians with hepatitis C genotype 1. Antiviral Res.

[22] Yu ML, Huang CF, Huang JF, Chang NC, Yang JF, Lin ZY, Chen SC (2011). Role of interleukin-28B polymorphisms in the treatment of hepatitis C virus genotype 2 infection in Asian patients. Hepatology.

[23] Mangia A, Thompson AJ, Santoro R, Piazzolla V, Tillmann HL, Patel K (2010). An IL28B polymorphism determines treatment response of hepatitis C virus genotype 2 or 3 patients who do not achieve a rapid virologic response. Gastroenterology.

[24] Sarrazin C, Susser S, Doehring A, Lange CM, Müller T, Schlecker C (2011). Importance of IL28B gene polymorphisms in hepatitis C virus genotype 2 and 3 infected patients. J Hepatol.

[25] Huang CF, Yeh ML, Hsieh MH, Hsieh MY, Lin ZY, Chen SC, Wang LY (2013). Clinical utility of host genetic IL-28B variants in hepatitis C virus genotype 1 Asian patients retreated with pegylated interferon plus ribavirin. J Gastroenterol Hepatol.

[26] Huang CF, Dai CY, Yeh ML, Huang JF, Huang CI, Hsieh MY (2013). Virological predictors of response to retreatment in hepatitis C genotype 2 infected patients. PLoS ONE.

[27] Yu M-L, Chuang W-L (2015). New treatments for HCV: perspective from Asia. Clin Liver Dis.

[28] Feld JJ, Kowdley KV, Coakley E (2014). Treatment of HCV with ABT-450/r-ombitasvir and dasabuvir with ribavirin. N Engl J Med.

[29] Afdhal N, Reddy KR, Nelson DR, Lawitz E, Gordon SC, Schiff E, Nahass R (2014). Ledipasvir and sofosbuvir for previously treated HCV genotype 1 infection. N Engl J Med.

[30] Afdhal N, Zeuzem S, Kwo P (2014). Ledipasvir and sofosbuvir for untreated HCV genotype 1 infection. N Engl J Med.

[31] George SL, Bacon BR, Brunt EM, Mihindukulasuriya KL, Hoffmann J, Di Bisceglie AM (2009). Clinical, virological, histologic, and biochemical outcomes after successful HCV therapy: a 5-year follow-up of 150 patients. Hepatology.

[32] Lindsay KL, Trepo C, Heintges T, Shiffman ML, Gordon SC, Hoels JC (2001). A randomized, double-blind trial comparing pegylated interferon alfa-2b to interferon alfa-2b as initial treatment for chronic Hepatitis C. Hepatology.

[33] Manns MP, McHutchison JG, Gordon SC (2001). Peginterferon alfa-2b plus ribavirin compared with interferon alfa-2b plus ribavirin for initial treatment of chronic hepatitis C: a randomised trial. Lancet.

[34] RiDruejo R (2012). Predictors of response to chronic hepatitis C treatment. Fut Virol.

[35] Wedemeyer H, Dore GJ, Ward JW (2015). Estimates on HCV disease burden worldwide—filling the gaps. J Viral Hepatitis..

[36] Kowdley KV, Gordon SC, Reddy KR, Rossaro L, Bernstein DE, Lawitz E, Shiffman ML (2014). Ledipasvir and sofosbuvir for 8 or 12 weeks for chronic HCV without cirrhosis. N Engl J Med.

[37] Charlton M, Gane E, Manns MP, Brown RS, Curry MP, Kwo PY (2015). Sofosbuvir and ribavirin for treatment of compensated recurrent hepatitis C virus infection after liver transplantation. Gastroenterology.

[38] Curry MP, Forns X, Chung RT, Terrault NA, Brown R, Fenkel JM (2015). Sofosbuvir and ribavirin prevent recurrence of HCV infection after liver transplantation: an open-label study. Gastroenterology.

[39] Poordad F, Hezode C, Trinh R, Kowdley KV, Zeuzem S, Agarwal K, Shiffman ML (2014). ABT-450/r-ombitasvir and dasabuvir with ribavirin for hepatitis C with cirrhosis. N Engl J Med.

[40] Zeuzem S, Jacobson IM, Baykal T (2014). Retreatment of HCV with ABT-450/r-ombitasvir and dasabuvir with ribavirin. N Engl J Med.

[41] Hezode C, Asselah T, Reddy KR (2015). Ombitasvir plus paritaprevir plus ritonavir with or without ribavirin in treatment-naive and treatment-experienced patients with genotype 4 chronic hepatitis C virus infection (PEARL-I): a randomised, open-label trial. Lancet.

[42] Molina JM, Orkin C, Iser DM (2015). Sofosbuvir plus ribavirin for treatment of hepatitis C virus in patients co-infected with HIV (PHOTON-2): a multicentre, open-label, non-randomised, phase 3 study. Lancet.

[43] Lim SG (2015). Chronic hepatitis C genotype 1 treatment roadmap for resource constrained settings. World J Gastroenterol.

[44] Zeuzem S, Andreone P, Pol S, Lawitz E, Diago M, Roberts S (2011). Telaprevir for retreatment of HCV infection. N Engl J Med.

[45] Manns M, Marcellin P, Poordad F, de Araujo ES, Buti M, Horsmans Y, Janczewska E, Villamil F, Scott J, Peeters M, Lenz O, Ouwerkerk-Mahadevan S, De La Rosa G, Kalmeijer R, Sinha R, Beumont-Mauviel M (2014). Simeprevir with pegylated interferon alfa 2a or 2b plus ribavirin in treatment-naive patients with chronic hepatitis C virus genotype 1 infection (QUEST-2): a randomised, double-blind, placebo-controlled phase 3 trial. Lancet.

[46] Jacobson IM, Dore GJ, Foster GR, Fried MW, Radu M, Rafalsky VV, Moroz L, Craxi A, Peeters M, Lenz O, Ouwerkerk-Mahadevan S, De La Rosa G, Kalmeijer R, Scott J, Sinha R, Beumont-Mauviel M (2014). Simeprevir with pegylated interferon alfa 2a plus ribavirin in treatment-naive patients with chronic hepatitis C virus genotype 1 infection (QUEST-1): a phase 3, randomised, double-blind, placebo-controlled trial. Lancet.

[47] Lawitz E, Sulkowski MS, Ghalib R, Rodriguez-Torres M, Younossi ZM, Corregidor A, DeJesus E, Pearlman B, Rabinovitz M, Gitlin N, Lim JK, Pockros PJ, Scott JD, Fevery B, Lambrecht T, Ouwerkerk-Mahadevan S, Callewaert K, Symonds WT, Picchio G, Lindsay KL, Beumont M, Jacobson IM (2014). Simeprevir plus sofosbuvir, with or without ribavirin, to treat chronic infection with hepatitis C virus genotype 1 in non-responders to pegylated interferon and ribavirin and treatment-naive patients: the COSMOS randomised study. Lancet.

[48] Reddy KR, Zeuzem S, Zoulim F, Weiland O, Horban A, Stanciu C, Villamil FG, Andreone P, George J, Dammers E, Fu M, Kurland D, Lenz O, Ouwerkerk-Mahadevan S, Verbinnen T, Scott J, Jessner W (2015). Simeprevir versus telaprevir with peginterferon and ribavirin in previous null or partial responders with chronic hepatitis C virus genotype 1 infection (ATTAIN): a randomised, double-blind, non-inferiority phase 3 trial. Lancet Infect Dis.

[49] Fellay J, Thompson AJ, Ge D, Gumbs CE, Urban TJ, Shianna KV, Little LD, Qiu P, Bertelsen AH, Watson M, Warner A, Muir AJ, Brass C, Albrecht J, Sulkowski M, McHutchison JG, Goldstein DB (2010). ITPA gene variants protect against anaemia in patients treated for chronic hepatitis C. Nature.

[50] Muir AJ, Poordad FF, McHutchison JG, Shiffman ML, Berg T, Ferenci P, Heathcote EJ, Pawlotsky JM, Zeuzem S, Reesink HW, Dusheiko G, Martin EC, George S, Kauffman RS, Adda N (2011). Retreatment with telaprevir combination therapy in hepatitis C patients with well-characterized prior treatment response. Hepatology.

[51] Thompson AJ, McHutchison JG (2012). Will IL28B polymorphism remain relevant in the era of direct-acting antiviral agents for hepatitis C virus?. Hepatology.

[52] Gane EJ, Roberts SK, Stedman CA, Angus PW, Ritchie B, Elston R, Ipe D, Morcos PN, Baher L, Najera I, Chu T, Lopatin U, Berrey MM, Bradford W, Laughlin M, Shulman NS, Smith PF (2010). Oral combination therapy with a nucleoside polymerase inhibitor (RG7128) and danoprevir for chronic hepatitis C genotype 1 infection (INFORM-1): a randomised, double-blind, placebo-controlled, dose-escalation trial. Lancet.

[53] Lawitz EJ, Gruener D, Hill JM, Marbury T, Moorehead L, Mathias A, Cheng G, Link JO, Wong KA, Mo H, McHutchison JG, Brainard DM (2012). A phase 1, randomized, placebo-controlled, 3-day, dose-ranging study of GS-5885, an NS5A inhibitor, in patients with genotype 1 hepatitis C. J Hepatol.

[54] Lawitz E, Poordad FF, Pang PS, Hyland RH, Ding X, Mo H, Symonds WT, McHutchison JG, Membreno FE (2014). Sofosbuvir and ledipasvir fixed-dose combination with and without ribavirin in treatment-naive and previously treated patients with genotype 1 hepatitis C virus infection (LONESTAR): an open-label, randomised, phase 2 trial. Lancet.

[55] Mizokami M, Yokosuka O, Takehara T, Sakamoto N, Korenaga M, Mochizuki H, Nakane K, Enomoto H, Ikeda F, Yanase M, Toyoda H, Genda T, Umemura T, Yatsuhashi H, Ide T, Toda N, Nirei K, Ueno Y, Nishigaki Y, Betular J, Gao B, Ishizaki A, Omote M, Mo H, Garrison K, Pang PS, Knox SJ, Symonds WT, McHutchison JG, Izumi N, Omata M (2015). Ledipasvir and sofosbuvir fixed-dose combination with and without ribavirin for 12 weeks in treatment-naive and previously treated Japanese patients with genotype 1 hepatitis C: an open-label, randomised, phase 3 trial. Lancet Infect Dis.

[56] Charlton M, Everson GT, Flamm SL, Kumar P, Landis C, Brown RS (2015). Ledipasvir and sofosbuvir plus ribavirin for treatment of HCV infection in patients with advanced liver disease. Gastroenterology.

[57] Chayama K, Notsumata K, Kurosaki M, Sato K, Rodrigues L, Setze C, Badri P, Pilot-Matias T, Vilchez RA, Kumada H (2015). Randomized trial of interferon- and ribavirin-free ombitasvir/paritaprevir/ritonavir in treatment-experienced hepatitis C virus-infected patients. Hepatology.

[58] Ferenci P, Bernstein D, Lalezari J, Cohen D, Luo Y, Cooper C, Tam E, Marinho RT, Tsai N, Nyberg A, Box TD, Younes Z, Enayati P, Green S, Baruch Y, Bhandari BR, Caruntu FA, Sepe T, Chulanov V, Janczewska E, Rizzardini G, Gervain J, Planas R, Moreno C, Hassanein T, Xie W, King M, Podsadecki T, Reddy KR (2014). PEARL-III study; PEARL-IV study. ABT-450/r-ombitasvir and dasabuvir with or without ribavirin for HCV. N Engl J Med.

[59] Lok AS, Gardiner DF, Lawitz E, Martorell C, Everson GT, Ghalib R, Reindollar R, Rustgi V, McPhee F, Wind-Rotolo M, Persson A, Zhu K, Dimitrova DI, Eley T, Guo T, Grasela DM, Pasquinelli C (2012). Preliminary study of two antiviral agents for hepatitis C genotype 1. N Engl J Med.

[60] Kumada H, Suzuki Y, Ikeda K, Toyota J, Karino Y, Chayama K, Kawakami Y, Ido A, Yamamoto K, Takaguchi K, Izumi N, Koike K, Takehara T, Kawada N, Sata M, Miyagoshi H, Eley T, McPhee F, Damokosh A, Ishikawa H, Hughes E (2014). Daclatasvir plus asunaprevir for chronic HCV genotype 1b infection. Hepatology.

[61] Fujii Y, Uchida Y, Mochida S (2015). Drug-induced immunoallergic hepatitis during combination therapy with daclatasvir and asunaprevir. Hepatology.

[62] Manns M, Pol S, Jacobson IM, Marcellin P, Gordon SC, Peng CY, Chang TT (2014). All-oral daclatasvir plus asunaprevir for hepatitis C virus genotype 1b: a multinational, phase 3, multicohort study. Lancet.

[63] Suda G, Kudo M, Nagasaka A, Furuya K, Yamamoto Y, Kobayashi T, Shinada K, Tateyama M, Konno J, Tsukuda Y, Yamasaki K, Kimura M, Umemura M, Izumi T, Tsunematsu S, Sato F, Terashita K, Nakai M, Horimoto H, Sho T, Natsuizaka M, Morikawa K, Ogawa K, Sakamoto N (2016) Efficacy and safety of daclatasvir and asunaprevir combination therapy in chronic hemodialysis patients with chronic hepatitis C. J Gastroenterol. doi:10.1007/s00535-016-1162-8 10.1007/s00535-016-1162-8 26768604

[64] Everson GT, Sims KD, Rodriguez-Torres M, Hézode C, Lawitz E, Bourlière M, Loustaud-Ratti V, Rustgi V, Schwartz H, Tatum H, Marcellin P, Pol S, Thuluvath PJ, Eley T, Wang X, Huang SP, McPhee F, Wind-Rotolo M, Chung E, Pasquinelli C, Grasela DM, Gardiner DF (2014). Efficacy of an interferon- and ribavirin-free regimen of daclatasvir, asunaprevir, and BMS-791325 in treatment-naive patients with HCV genotype 1 infection. Gastroenterology.

[65] Lawitz E, Gane E, Pearlman B, Tam E, Ghesquiere W, Guyader D, Alric L, Bronowicki JP, Lester L, Sievert W, Ghalib R, Balart L, Sund F, Lagging M, Dutko F, Shaughnessy M, Hwang P, Howe AY, Wahl J, Robertson M, Barr E, Haber B (2015). Efficacy and safety of 12 weeks versus 18 weeks of treatment with grazoprevir (MK-5172) and elbasvir (MK-8742) with or without ribavirin for hepatitis C virus genotype 1 infection in previously untreated patients with cirrhosis and patients with previous null response with or without cirrhosis (C-WORTHY): a randomised, open-label phase 2 trial. Lancet.

[66] Roth D, Nelson DR, Bruchfeld A, Liapakis A, Silva M, Monsour H (2015). Grazoprevir plus elbasvir in treatment-naive and treatment-experienced patients with hepatitis C virus genotype 1 infection and stage 4–5 chronic kidney disease (the C-SURFER study): a combination phase 3 study. Lancet.

[67] Hirotsu Y, Kanda T, Matsumura H, Moriyama M, Yokosuka O, Omata M (2015). HCV NS5A resistance-associated variants in a group of real-world Japanese patients chronically infected with HCV genotype 1b. Hepatol Int.

[68] Zeuzem S, Dusheiko GM, Salupere R, Mangia A, Flisiak R, Hyland RH, Illeperuma A (2014). Sofosbuvir and ribavirin in HCV genotypes 2 and 3. N Engl J Med.

[69] Gane EJ, Stedman CA, Hyland RH (2013). Nucleotide polymerase inhibitor sofosbuvir plus ribavirin for hepatitis C. N Engl J Med.

[70] Lawitz E, Mangia A, Wyles D (2013). Sofosbuvir for previously untreated chronic hepatitis C infection. N Engl J Med.

[71] Jacobson IM, Gordon SC, Kowdley KV (2013). Sofosbuvir for hepatitis C genotype 2 or 3 in patients without treatment options. N Engl J Med.

[72] Omata M, Nishiguchi S, Ueno Y, Mochizuki H, Izumi N, Ikeda F, Toyoda H, Yokosuka O, Nirei K, Genda T, Umemura T, Takehara T, Sakamoto N, Nishigaki Y, Nakane K, Toda N, Ide T, Yanase M, Hino K, Gao B, Garrison KL, Dvory-Sobol H, Ishizaki A, Omote M, Brainard D, Knox S, Symonds WT, McHutchison JG, Yatsuhashi H, Mizokami M (2014). Sofosbuvir plus ribavirin in Japanese patients with chronic genotype 2 HCV infection: an open-label, phase 3 trial. J Viral Hepatol.

[73] Sulkowski MS, Gardiner DF, Rodriguez-Torres M (2014). Daclatasvir plus sofosbuvir for previously treated or untreated chronic HCV infection. N Engl J Med.

[74] Gane EJ, Hyland RH, Yang Y, Svarovskaia E, Pang PS, McHutchison JG, et al. Ledipasvir/Sofosbuvir single tablet regimen is effective in patients with HCV Genotype 2 Infection. In: 15th international symposium on viral hepatitis and liver disease (ISVHLD), Berlin, Germany, on 26–28 Jun 2015. http://www.natap.org/2015/HCV/070215_02.htm. Accessed on 11 Feb 2016

[75] Everson GT, Tran TT, Towner WJ, et al. Safety and efficacy of treatment with the interferon-free, ribavirin-free combination of sofosbuvir + GS-5816 for 12 weeks in treatment naive patients with genotype 1-6 HCV patients (Abstract). J Hepatol 2014;60(Suppl.):S46

[76] Nelson DR, Cooper JN, Lalezari JP et al. All-oral 12-week combination treatment with daclatasvir (DCV) and sofosbuvir (SOF) in patients infected with HCV genotype (GT) 3: ALLY-3 phase 3 study. In: Abstract LB-3. 65th Annual Meeting of the American Association for the Study of Liver Diseases (AASLD). 7–11 Nov 2014; Boston, MA

[77] Ruane P, Ain D, Meshrekey R, Stryker R. Sofosbuvir plus ribavirin in the treatment of chronic HCV genotype 4 infection in patients of Egyptian ancestry. In: 64th Annual Meeting of the American Association for the Study of Liver Diseases (AASLD). 1–5 Nov 2013; Washington DC

[78] Esmat GE, Shiha G, Omar RF et al. Sofosbuvir plus ribavirin in the treatment of egyptian patients with chronic genotype 4 HCV infection. In: Abstract 959. 65th Annual Meeting of the American Association for the Study of Liver Diseases (AASLD). 7–11 Nov 2014; Boston, MA

[79] Molina JM, Orkin C, Iser DM et al. All-oral therapy with sofosbuvir plus ribavirin for the treatment of HCV genotypes 1, 2, 3 and 4 infection in patients co-infected with HIV (PHOTON-2). In: Abstract MOAB0105LB. 20th International AIDS Conference. 20–25 July 2014; Melbourne, Australia

[80] Kapoor R, Kohli A, Sidharthan S et al. all-oral treatment for genotype 4 chronic hepatitis C infection with sofosbuvir and ledipasvir: interim results from the NIAID SYNERGY trial. In: Abstract 240. 65th Annual Meeting of the American Association for the Study of Liver Diseases (AASLD). 7–11 Nov 2014; Boston, MA

[81] Pol S, Reddy KR, Baykal T et al. Interferon-free regimens of ombitasvir and ABT-450/r with or without ribavirin in patients with HCV genotype 4 infection: PEARL-I study results. In: Abstract 1928. 65th Annual Meeting of the American Association for the Study of Liver Diseases (AASLD). 7–11 Nov 2014; Boston, MA

[82] Gane EJ, Hyland RH, An D, Svarovskaia ES, Pang PS, Symonds WT (2014). High efficacy of LDV/SOF regimens for 12 weeks for patients with HCV genotype 3 or 6 infection. Hepatology.

[83] Moreno C, Hezode C, Marcellin P et al. Simeprevir with peginterferon/ribavirin for treatment of chronic HCV genotype 4 infection in treatment-naïve or -experienced patients: interim results of a phase III trial. In: Abstract 60. Hep DAART. 8–12 Dec 2013; Big Island, Hawaii

[84] Zeuzem S, Ghalib R, Reddy KR (2015). Grazoprevir–Elbasvir combination therapy for treatment-naive cirrhotic and noncirrhotic patients with chronic HCV genotype 1, 4, or 6 infection: a randomized trial. Ann Intern Med.

[85] Everson G et al. Safety and efficacy of treatment with the interferon-free, ribavirin-free combination of sofosbuvir + GS-5816 for 12 weeks in treatment naive patients with genotype 1–6 HCV infection. In: 49th Annual Meeting of the European Association for the Study of the Liver (EASL), abstract O111, London, 2014

[86] Clinicaltrials.gov NCT02201940; Gilead press release. Sept 21, 2015

[87] Hill A, Khoo S, Fortunak J (2014). Minimum costs for producing hepatitis C direct-acting antivirals for use in large-scale treatment access programs in developing countries. Clin Infect Dis.

[88] Kohli A, Osinusi A, Sims Z (2015). Virological response after 6 week triple-drug regimens for hepatitis C: a proof-of-concept phase 2A cohort study. Lancet.

[89] Qureshi H, Bile KM, Jooma R, Alam SE, Afridi HU (2010). Prevalence of hepatitis B and C viral infections in Pakistan: findings of a national survey appealing for effective prevention and control measures. East Mediterr Health J.

[90] Janjua NZ, Hamza HB, Islam M, Tirmizi SF, Siddiqui A, Jafri W (2010). Health care risk factors among women and personal behaviors among men explain the high prevalence of hepatitis C virus infection in Karachi, Pakistan. J Viral Hepat.

[91] Mumtaz K, Hamid SS, Moatter T, Abid S, Shah HA, Jafri W (2008). Distribution of hepatitis C virus genotypes and its response to treatment in Pakistani patients. Saudi Med J.

[92] Abergel A, Loustaud-Ratti V, Metivier S, Jiang D, Kersey K, Knox SJ (2015). Ledipasvir/sofosbuvir treatment results in high SVR rates in patients with chronic genotype 4 and 5 HCV infection. J Hepatol.

[93] Wehmeyer MH, Jordan S, Eißing C, Hartl J, Stohr A, Petersen J (2015). Sofosbuvir in combination with peginterferon and ribavirin for patients chronically infected with hepatitis C virus genotype 4: “real-life” experience of two large viral hepatitis centers in northern Germany. J Hepatol.

[94] Reddy R, Lim JK, Kuo A, Di Bisceglie AM, Vargas HE, Galati JS (2015). all-oral HCV therapy is safe and effective in patients with decompensated cirrhosis: interim report from the HCV-target real world experience. J Hepatol.

[95] Samuel D, Manns M, Forns X, Flamm SL, Reddy KR, Denning J (2015). Ledipasvir/sofosbuvir with ribavirin is safe in >600 decompensated and post liver transplantation patients with HCV infection: an integrated safety analysis of the solar 1 and solar 2 trials D. J Hepatol.

[96] Waked I, Doss W, El-Sayed MH, Razavi H, Shiha G, Yosry A, Esmat G (2014). The current and future disease burden of chronic hepatitis C virus infection in Egypt. Arab J Gastroenterol.

[97] Doss W, Esmat G, El Serafy M, El Sayed M, Hassany M, Aymen Yousry A, El Akel W, and waked I:(National Committee for Control of Viral Hepatitis (NCCVH), Ministry of Health and Population, Egypt): Interim analysis for Sofosbuvir National treatment Program in Egypt, 15th International symposium on viral hepatitis and liver (ISVHL), Poster 149, 26–28 June 2015

[98] Gani RA, Hasan I, Sanityoso A, Lesmana CRA, Waspodo AS, Siregar L (2014). National Consensus of Hepatitis C Infection Management in Indonesia.

[99] Akbar N, Sulaiman A, Hasan I, Lesmana LA, Gani RA, Sanityoso A (2009). Efficacy and safety of In-Asia-manufactured Interferon α -2b in combination with Ribavirin for therapy of naïve chronic hepatitis C patients: a multicenter, prospective, open-label trial. Indones J Gastroenterol Hepatol Dig Endosc.

[100] Juniastuti K, Wibowo BP, Wibawa IDN, Utsumi T, Mustika S, Amin M (2014). Interleukin-28B polymorphisms and response of chronic hepatitis C patients from Indonesia to Pegylated Interferon/Ribavirin treatment. J Clin Microbiol.

[101] Navasa M, Forns X (2007). Antiviral therapy in HCV decompensated cirrhosis: to treat or not to treat. J Hepatol.

[102] Berenguer M, Palau A, Aguilera V, Rayón JM, Juan FS, Prieto M (2008). Clinical benefits of antiviral therapy in patients with recurrent hepatitis C following liver transplantation. Am J Transplant.

[103] Kwo PY, Mantry PS, Coakley E, Te HS, Vargas HE, Brown R (2014). An interferon-free antiviral regimen for HCV after liver transplantation. N Engl J Med.

[104] Foster GR, McLauchlan J, W. Irving, M. Cheung, B. Hudson, S. Verma, K. Agarwal, HCV Research UK EAP Group. Treatment of decompensated HCV cirrhosis in patients with diverse genotypes: 12 weeks sofosbuvir and NS5A inhibitors with/without ribavirin is effective in HCV Genotypes 1 and 3. EASL - The International Liver Congress 2015 50th Annual Meeting of the European Association for the Study of the Liver, Vienna, Austria, 22–26 April 2015

[105] Afdhal N, Everson G, McCaughan G (2014). Sofosbuvir and ribavirin for the treatment of chronic HCV with cirrhosis and portal hypertension with and without decompensation: early virologic response and safety. J Hepatol.

[106] Reddy KR, Lim J, Kuo A, et al. all-oral HCV therapy is safe and effective in patients with decompensated cirrhosis: report from HCV-TARGET. EASL - the international liver congress 2015 50th annual meeting of the European Association for the Study of the Liver, Vienna, Austria, 22–26 April 2015

[107] Bashar A, Pungpapong S (2014). The use of simeprevir and sofosbuvir to treat HCV G1 in the liver transplant setting: the experience in 3 US Centres. Hepatology.

[108] Reddy KR, Everson GT, Flamm SL, Denning JM, Arterburn S, Brandt-Sarif T (2014). Ledipasvir/sofosbuvir with ribavirin for the treatment of HCV in patients with post-transplant recurrence: preliminary results of a prospective, multicenter study. Hepatology.

[109] Jensen DM, O’Leary JG, Pockros P (2014). Safety and efficacy of sofosbuvir-containing regimens for hepatitis C: real-world experience in a diverse, longitudinal observational cohort. J Hepatol.

[110] Brown RS, Reddy KRJ, O’Leary JG, Kuo A, Morelli G, Stravitz RT, Durand CM (2014). Safety and efficacy of new DAA-based therapy for hepatitis C post-transplant: interval results from the HCV-TARGET longitudinal, observational study. Hepatology.

[111] Forns X, Berenguer M, Herzer K, et al. On-treatment virologic response and tolerability of simeprevir, daclatasvir and ribavirin in patients with recurrent hepatitis C virus genotype 1b infection after orthotopic liver transplantation (OLT): interim data from the Phase II SATURN study. In: EASL - The International Liver Congress 2015 50th Annual Meeting of the European Association for the Study of the Liver, Vienna, Austria, 22–26 April

[112] Forns X, Charlton M, Denning J (2015). Sofosbuvir compassionate use program for patients with severe recurrent hepatitis C after liver transplantation. Hepatology.

[113] Leroy V, Dumortier J, Coilly A, Sebagh M, Fougerou-Leurenl C, Radenne S, Bolla D (2014). High rates of virological response and major clinical improvement during sofosbuvir and daclatasvir-based regimens for the treatment of fibrosing cholestatic HCV recurrence after liver transplantation: the ANRS CO23 CUPILT study. Hepatology.

[114] Coilly A, Fougerou C, De Ledinghen V, et al. The association of sofosbuvir and daclatasvir for treating severe recurrence of HCV infection after liver transplantation. EASL - The International Liver Congress 2015 50th Annual Meeting of the European Association for the Study of the Liver, Vienna, Austria, 22–26 April 2015

[115] Fissel RB, Bragg-Gresham JL, Woods JD, Jadoul M, Gillespie B, Heddewick SA (2004). Patterns of hepatitis C prevalence and seroconversion in hemodialysis units from three continents: the DOPPS. Kidney Int.

[116] Kidney Disease: Improving Global Outcomes (2008) KDIGO clinical practice guidelines for the prevention, diagnosis, evaluation, and treatment of hepatitis C in chronic kidney disease. Kidney Int Suppl (109):S1-99 10.1038/ki.2008.81 18382440

[117] Butt AA, Skanderson M, McGinnis KA, Ahuja T, Bryce CL, Barnato AE (2007). Impact of hepatitis C virus infection and other comorbidities on survival in patients on dialysis. J Viral Hepat.

[118] Henderson WA, Shankar R, Gill JM, Kim KH, Ghany MG, Skanderson M (2010). Hepatitis C progressing to hepatocellular carcinoma: the HCV dialysis patient in dilemma. J Viral Hepat.

[119] Pineda JA, Romero-Gómez M, Díaz-García F, Girón-González JA, Montero JL, Torre-Cisneros J (2005). HIV co-infection shortens the survival of patients with hepatitis C virus-related decompensated cirrhosis. Hepatology.

[120] Macías J, Márquez M, Téllez F, Merino D, Jiménez-Aguilar P, López-Cortés LF (2013). Risk of liver decompensation among HIV/hepatitis C virus-co-infected individuals with advanced fibrosis: implications for the timing of therapy. Clin Infect Dis.

[121] Alves JP, Peres S, Borges F, Miranda AC, Baptista T, Ventura F (2014). Risk of liver decompensation assessed in HIV/HCV co-infected individuals with advanced liver fibrosis: a faster countdown experience. J Int AIDS Soc.

[122] Osinusi A, Townsend K, Kohli A, Nelson A, Seamon C, Meissner EG (2015). Virologic response following combined ledipasvir and sofosbuvir administration in patients with HCV genotype 1 and HIV co-infection. JAMA.

[123] Coppola N, Martini S, Pisaturo M, Sagnelli C, Filippini P, Sagnelli E (2015). Treatment of chronic hepatitis C in patients with HCV/HIV co-infection. World J Virol.

[124] Panel on Antiretroviral Guidelines for Adults and Adolescents. Guidelines for the use of antiretroviral agents in HIV-1-infected adults and adolescents. Department of Health and Human Services. Available at http://www.aidsinfo.nih.gov/ContentFiles/AdultandAdolescentGL.pdf. Accessed 25 April 2015

[125] Sharma SA, Feld JJ (2015). Management of HCV in cirrhosis-a rapidly evolving landscape. Curr Gastroenterol Rep.

[126] Limketkai BN, Mehta SH, Sutcliffe CG (2012). Relationship of liver disease stage and antiviral therapy with liver-related events and death in adults co-infected with HIV/HCV. JAMA.

[127] Mira JA, Rivero-Juárez A, López-Cortes LF (2013). Benefits from sustained virologic response to pegylated interferon plus ribavirin in HIV/hepatitis C virus co-infected patients with compensated cirrhosis. Clin Infect Dis.

[128] Nunez M, Marino A, Miralles C, Berdun MA, Sola J, Hernandez-Burruezo JJ (2007). Baseline serum hepatitis C virus (HCV) RNA level and response at week 4 are the best predictors of relapse after treatment with pegylated interferon plus ribavirin in HIV/HCV-co-infected patients. J Acquir Immune Defic Syndr.

[129] Martin-Carbonero L, Nunez M, Marino A, Alcocer F, Bonet L, Garcia-Samaniego J (2008). Undetectable hepatitis C virus RNA at week 4 as predictor of sustained virological response in HIV patients with chronic hepatitis C. AIDS.

[130] Berenguer J, Alvarez-Pellicer J, Martin PM, Lopez-Aldeguer J, Von-Wichmann MA, Quereda C (2009). Sustained virological response to interferon plus ribavirin reduces liver-related complications and mortality in patients co-infected with human immunodeficiency virus and hepatitis C virus. Hepatology.

[131] Pineda JA, Caruz A, Rivero A, Neukam K, Salas I, Camacho A (2010). Prediction of response to pegylated interferon plus ribavirin by IL28B gene variation in patients co-infected with HIV and hepatitis C virus. Clin Infect Dis.

[132] Clausen LN, Weis N, Astvad K, Schonning K, Fenger M, Krarup H (2011). Interleukin-28B polymorphisms are associated with hepatitis C virus clearance and viral load in a HIV-1-infected cohort. J Viral Hepat.

[133] Rallon NI, Naggie S, Benito JM, Medrano J, Restrepo C, Goldstein D (2010). Association of a single nucleotide polymorphism near the interleukin-28B gene with response to hepatitis C therapy in HIV/hepatitis C virus-co-infected patients. AIDS.

[134] Liu CJ, Chuang WL, Lee CM, Yu ML, Lu SN, Wu SS (2009). Peginterferon alfa-2a plus ribavirin for the treatment of dual chronic infection with hepatitis B and C viruses. Gastroenterology.

[135] Potthoff A, Berg T, Wedemeyer H (2009). Late hepatitis B virus relapse in patients co-infected with hepatitis B virus and hepatitis C virus after antiviral treatment with pegylated interferon-a2b and ribavirin. Scand J Gastroenterol.

[136] Yeh ML, Hung CH, Huang JF, Liu CJ, Lee CM, Dai CY (2011). Long-term effect of interferon plus ribavirin on hepatitis B surface antigen seroclearance in patients dually infected with hepatitis B and C viruses. PLoS ONE.

[137] Yu ML, Lee CM, Chuang WL, Lu SN, Dai CY, Huang JF (2010). HBsAg profiles in patients receiving peginterferon alfa-2a plus ribavirin for the treatment of dual chronic infection with hepatitis B and C viruses. J Infect Dis.

