# Supplementary material for: APASL consensus statements and recommendation on treatment of hepatitis C
Source: Hepatol Int. 2016 Apr 29;10(5):702–26. doi: 10.1007/s12072-016-9717-6 (PMC5003907; doi:10.1007/s12072-016-9717-6)
Supplement: Supplementary file 1 — Supplementary material 1 (DOCX 31 kb) [file 12072_2016_9717_MOESM1_ESM.docx]

**Suppl. Table 1.** Grading of evidence and recommendations (adapted from the GRADE system) [1,2].

| **Grading of evidence** | **Notes** | **Symbol** |
| --- | --- | --- |
| High quality | Further research is very unlikely to change our confidence in the estimate of effect | A |
| Moderate quality | Further research is likely to have an important impact on our confidence in the estimate of effect and may change the estimate | B |
| Low or very low quality | Further research is very likely to have an important impact on our confidence in the estimate of effect and is likely to change the estimate. Any estimate of effect is uncertain | C |
| **Grading of recommendation** | **Notes** | **Symbol** |
| Strong recommendation warranted | Factors influencing the strength of the recommendation included the quality of the evidence, presumed patient important outcomes, and cost | 1 |
| Weaker recommendation | Variability in preferences and values, or more uncertainty: more likely a weak recommendation is warranted. Recommendation is made with less certainty; higher cost or resource consumption | 2 |

Ref

1. Guyatt GH, Oxman AD, Vist GE, Kunz R, Falck-Ytter Y, Alonso-Coello P, et al.GRADE: an emerging consensus on rating quality of evidence and strength of recommendations. BMJ 2008;336:924–926

2. Schunemann HJ, Oxman AD, Brozek J, Glasziou P, Jaeschke R, Vist GE, et al. Grading quality of evidence and strength of recommendations for diagnostic tests and strategies. BMJ 2008;336:1106–1110

**Suppl. Table 2 Recommended treatment regimens for HCV-patients with treatment-naïve or previously treated with peginterferon plus ribavirin (Compared with AASLD guideline)**

|  | Drug (daily dose) | Duration (weeks) | APASL | AASLD |
| --- | --- | --- | --- | --- |
| GT-1 | Ledipasvir (90 mg)/Sofosbuvir (400 mg) | 12 | A1 | A-I |
| GT-2 | Sofosbuvir (400 mg) and RBV | 12 | A1 | A-I |
| GT-2 | Daclatasvir (60 mg) and Sofosbuvir (400 mg) | 12 | B1 |  |
| GT-2 with cirrhosis | Sofosbuvir (400 mg) and RBV | 16 | B2 | C-IIb |
| GT-2 with previous treatment | Sofosbuvir (400 mg) and RBV | 16 or 24 | A1 |  |
| GT-3 | Sofosbuvir (400 mg) and RBV | 24 | A1 | A-I |
| GT-3 without cirrhosis | Daclatasvir (60 mg) and Sofosbuvir (400 mg) | 12 | A1 |  |
| GT-3 with cirrhosis | Daclatasvir (60 mg) and Sofosbuvir (400 mg)+ RBV | 24 | B2 |  |
| GT-3 with previous treatment (no cirrhosis) | Daclatasvir (60 mg) and Sofosbuvir (400 mg) | 12 | A1 |  |
| GT-3 with previous treatment (cirrhosis) | Daclatasvir (60 mg) and Sofosbuvir (400 mg)+ RBV | 24 | B2 |  |
| GT-4 | Ledipasvir (90 mg)/Sofosbuvir (400 mg) | 12 | A1 | - |
| GT-4 | Ledipasvir (90 mg)/Sofosbuvir (400 mg) | 24 | A1  (for cirrhotics) | B-IIb |
| GT-4 | Abbvie 2D plus RBV | 12 | B1 | B-I |
| GT-4 | Abbvie 2D plus RBV | 24 | B1 | - |
| GT-4 | Daclatasvir (60 mg)/Sofosbuvir (400 mg) | 12-24 | B2 | - |
| GT-5/GT-6 | Ledipasvir (90 mg)/Sofosbuvir (400 mg)±RBV | 12 | B1 | B-IIa |
| GT-5/GT-6 | Daclatasvir (60 mg)/Sofosbuvir (400 mg) | 12-24 | B1 | - |

GT, genotype; RBV, ribavirin

Ref. AASLD/IDSA HCV Guidance Panel. Hepatitis C guidance: AASLD-IDSA recommendations for testing, managing, and treating adults infected with hepatitis C virus. Hepatology. 2015 Sep;62(3):932-54.
